# Misrepresentation and Nonadherence Regarding COVID-19 Public Health Measures

**DOI:** 10.1001/jamanetworkopen.2022.35837

**Published:** 2022-10-10

**Authors:** Andrea Gurmankin Levy, Alistair Thorpe, Laura D. Scherer, Aaron M. Scherer, Frank A. Drews, Jorie M. Butler, Nicole Burpo, Holly Shoemaker, Vanessa Stevens, Angela Fagerlin

**Affiliations:** 1 Department of Social and Behavioral Sciences, Middlesex Community College, Middletown, Connecticut; 2 Department of Population Health Sciences, University of Utah Spencer Fox Eccles School of Medicine, Salt Lake City; 3 Division of Cardiology, University of Colorado School of Medicine, Aurora; 4 Veterans Affairs (VA) Denver Center for Innovation, Denver, Colorado; 5 Department of Internal Medicine, University of Iowa School of Medicine, Iowa City; 6 Department of Psychology, University of Utah College of Social and Behavioral Science, Salt Lake City; 7 Salt Lake City VA Informatics Decision-Enhancement and Analytic Sciences (IDEAS) Center for Innovation, Salt Lake City, Utah; 8 Geriatrics Research, Education, and Clinical Center (GRECC), VA Salt Lake City Health Care System, Salt Lake City, Utah; 9 Department of Biomedical Informatics, University of Utah Spencer Fox Eccles School of Medicine, Salt Lake City; 10 Division of Geriatrics, Department of Internal Medicine, University of Utah Spencer Fox Eccles School of Medicine, Salt Lake City; 11 Department of Research, Office of Science Operations, American Heart Association, Dallas, Texas

## Abstract

**Importance:**

The effectiveness of public health measures implemented to mitigate the spread and impact of SARS-CoV-2 relies heavily on honesty and adherence from the general public.

**Objective:**

To examine the frequency of, reasons for, and factors associated with misrepresentation and nonadherence regarding COVID-19 public health measures.

**Design, Setting, and Participants:**

This survey study recruited a national, nonprobability sample of US adults to participate in an online survey using Qualtrics online panels (participation rate, 1811 of 2260 [80.1%]) from December 8 to 23, 2021. The survey contained screening questions to allow for a targeted sample of one-third who had had COVID-19, one-third who had not had COVID-19 and were vaccinated, and one-third who had not had COVID-19 and were unvaccinated.

**Main Outcomes and Measures:**

The survey assessed 9 different types of misrepresentation and nonadherence related to COVID-19 public health measures and the reasons underlying such behaviors. Additional questions measured COVID-19–related beliefs and behaviors and demographic characteristics.

**Results:**

The final sample included 1733 participants. The mean (SD) participant age was 41 (15) years and the sample predominantly identified as female (1143 of 1732 [66.0%]) and non-Hispanic White (1151 of 1733 [66.4%]). Seven hundred twenty-one participants (41.6%) reported misrepresentation and/or nonadherence in at least 1 of the 9 items; telling someone they were with or about to be with in person that they were taking more COVID-19 preventive measures than they actually were (420 of 1726 [24.3%]) and breaking quarantine rules (190 of 845 [22.5%]) were the most common manifestations. The most commonly endorsed reasons included wanting life to feel normal and wanting to exercise personal freedom. All age groups younger than 60 years (eg, odds ratio for those aged 18-29 years, 4.87 [95% CI, 3.27-7.34]) and those who had greater distrust in science (odds ratio, 1.14 [95% CI, 1.05-1.23]) had significantly higher odds of misrepresentation and/or nonadherence for at least 1 of the 9 items.

**Conclusions and Relevance:**

In this survey study of US adults, nearly half of participants reported misrepresentation and/or nonadherence regarding public health measures against COVID-19. Future work is needed to examine strategies for communicating the consequences of misrepresentation and nonadherence and to address contributing factors.

## Key Points

**Question:**

What are the prevalence of and reasons for misrepresentation and nonadherence regarding public health measures against COVID-19?

**Findings:**

In this national survey study of 1733 US adults, nearly half of participants reported misrepresentation and/or nonadherence regarding COVID-19 public health measures. The most common reasons included wanting life to feel normal and wanting to exercise personal freedom.

**Meaning:**

These findings suggest that misrepresentation and nonadherence regarding COVID-19 public health measures constitute a serious public health challenge.

## Teaser

This survey study explores the prevalence of, reasons for, and characteristics associated with misrepresentation and nonadherence regarding COVID-19 public health measures among survey respondents in the US.

## Introduction

SARS-CoV-2 was first identified in December 2019 and rapidly spread around the world, with more than 6 million people worldwide^1^ and more than 1 million in the US having died.^2^ In response, governments, organizations, school districts, and businesses worldwide implemented public health measures to mitigate the spread of the disease (eg, health screens, testing, quarantining, and vaccination requirements).

Public health measures have the potential to dramatically reduce the spread and impact of the disease,^3,4,5^ but their success depends on the public’s willingness to be honest about and adherent to these measures. However, these measures can involve significant psychological,^6^ social (eg, distancing from friends and family),^7,8^ financial (eg, loss of employment and/or income),^9^ and physical (eg, delayed health care appointments leading to delays in diagnosis and treatment)^10,11,12^ burdens that make adherence difficult.

Given the difficulty and costs associated with many public health measures, members of the public may exercise dishonesty and nonadherence regarding these measures. For example, people may withhold information about having COVID-19 during a health screening to allow them to attend their health care appointment or continue going to work. Reporting being vaccinated when one is not would allow a person to participate in an event restricted to those who are vaccinated or to avoid judgment from vaccinated friends.

Such scenarios are quite plausible given that it is well-established that people often misrepresent or do not disclose information in general,^13,14^ and specifically regarding their medical information and behaviors, particularly when it is embarrassing or potentially self-serving.^15,16,17^ For instance, previous research^16,17^ found that a substantial percentage of US adults withhold important information from their clinicians and that the reasons for doing so include concerns about being judged or lectured and not wanting to have to make a difficult change in behavior. In addition, in a national probability sample of adults receiving medical care for HIV,^15^ a substantial minority had sex without disclosing their positive HIV status.

Misrepresentation of COVID-19–related health information and nonadherence to public health measures may undermine the effectiveness of these measures. One of the few studies that examined this question early in the pandemic^18^ found that 55% of respondents reported some concealment of COVID-19 symptoms, and among those instructed to quarantine, 53% reported denying the need to do so when asked. Another study^19^ found that approximately 10% of full-time in-person employees reported going to work despite having a confirmed case of COVID-19 or a close contact with a confirmed case, and in a study of 69 people who were told to quarantine due to COVID-19 exposure or possible symptoms,^20^ nearly half (46%) broke quarantine rules.

These few studies were conducted very early in the pandemic, only examined a few types of misrepresentation or nonadherence, and/or did not explore the reasons for these behaviors. Therefore, our understanding of the prevalence of and reasons for these behaviors during the COVID-19 pandemic remains limited. To address these limits, the present study explored the prevalence of, reasons for, and characteristics associated with misrepresentation and nonadherence regarding COVID-19 public health measures.

## Methods

From December 8 to 23, 2021, we conducted an online survey of US adults that focused on their experiences and challenges throughout the COVID-19 pandemic. Qualtrics online panels were used to send targeted invitations to potential participants, and those interested began the survey. Qualtrics was used in part because of its access to a diversified cohort of US residents. Using an online platform also allowed for additional screening to ensure adequate representation. Specifically, screener questions at the beginning of the survey were used to select a sample in which one-third had had COVID-19, one-third had not had COVID-19 and were at least partially vaccinated (ie, received ≥1 dose of the Ad.26.COV2.S [Johnson & Johnson/Janssen], BNT162b2 [Pfizer], or mRNA-1273 [Moderna] vaccine), and one-third had not had COVID-19 and had not received any COVID-19 vaccinations. Multiple mechanisms were in place to ensure that the survey could not be completed by “bots” or cheaters (eMethods in the Supplement). The study was approved by the Institutional Review Board of the University of Utah. Consent to participate was given electronically by each participant before starting the survey. Participants were compensated based on the terms of their agreement with Qualtrics. The study followed the American Association for Public Opinion Research (AAPOR) reporting guideline.

### Survey

The survey was developed by the study team, which was composed of health services researchers and psychologists. In addition, pilot testing was conducted and feedback was sought from the lay public to assess the comprehensibility and mechanics of the survey (eMethods in the Supplement). The survey began by asking whether participants had ever had COVID-19 or symptoms that they thought might be COVID-19, had been vaccinated for COVID-19, and had ever been told to follow quarantine rules. Based on these responses, participants were presented with as many as 9 questions assessing misrepresentation and nonadherence that applied to them.

#### Misrepresentation Items

Participants indicated whether, during the COVID-19 pandemic, they did not mention that they thought they might have or knew they had COVID-19 to someone they were with or were about to be with in person (among those who thought they might have or knew they had COVID-19). The survey also assessed whether, throughout the pandemic, participants did not mention that they thought they might have or knew they had COVID-19 when being screened to enter a clinician’s office or a public place (among those who thought they might have or knew they had COVID-19), told someone they were with or were about to be with that they were taking more COVID-19 prevention measures than they actually were, told someone they were vaccinated for COVID-19 when they were not (among those who were not vaccinated), told someone they were not vaccinated when they were (among those who were vaccinated), and told someone that they did not have to quarantine when they were supposed to (among those who were told to quarantine).

#### Nonadherence Items

Participants were also asked whether, during the pandemic, they had ever avoided getting tested (among those who thought they might have COVID-19) and if they ever broke quarantine rules (among those who were told to quarantine).

#### Reasoning

If participants answered affirmatively to any of the misrepresentation or nonadherence items, they were asked to indicate yes or no to a list of reasons for the corresponding behavior. These reasons were consistent across similar items but differed where appropriate. They included items such as: “It’s no one else’s business,” “I didn’t want someone to judge or think badly of me,” “I wanted to exercise my freedom to do what I want,” and “I didn’t think COVID-19 was real.” Participants could say yes to all reasons that applied to them and type additional reasons for each item if desired.

#### Demographics

Participants also reported their religious beliefs (referred to hereinafter as *religiosity*), political party affiliation, political social beliefs, vaccine attitudes, where they obtained COVID-19 information, how often they wore a mask in a retail or grocery store, whether they have taken more or fewer COVID-19 prevention measures compared with those with whom they interact, belief in science,^21^ and conspiracy beliefs about COVID-19.^22^ Participants self-reported their age, gender identity, race and ethnicity, and location of residence (state and size of town or city). Race and ethnicity were included to allow us to describe the sample and because COVID-19 and its public health measures disproportionately impacted individuals from underserved populations. Finally, participants could provide feedback on the survey in an open text box.

### Statistical Analysis

To ensure a conservative estimate of misrepresentation and nonadherence among participants, for those who provided a comment in an open response opportunity that led us to question the validity of their affirmative response to a misrepresentation or nonadherence question, we did not include their affirmative response in the analyses. For instance, a few participants indicated that they did not mention thinking they might have or knowing they had COVID-19 in a health screen for a clinician appointment, but then wrote in an open response text box that they never left home while they had COVID-19.

Analyses were conducted in R Studio,^23^ version 1.4.1106 (R Program for Statistical Computing) with the use of multiple packages.^24,25,26^ We report demographic statistics, the percentage of participants who engaged in the 9 misrepresentation and nonadherence behaviors, and the reasons for these behaviors.

We used multiple logistic regression to conduct an exploratory analysis of the characteristics that were associated with whether or not participants reported misrepresentation or nonadherence in any of the 9 items (0, no reported misrepresentation or nonadherence; 1, reported misrepresentation or nonadherence in at least 1 of the 9 items). Separate models for each of the 9 items did not differ substantially from the overall model and are provided in the eTable in the Supplement). Significance was set at 2-sided α = .05. To account for multiple comparisons, *P* values were adjusted using Holm-Bonferroni correction.^27^

## Results

### Participants

Of the 2260 people who received the email invitation and accessed the survey, 1811 completed it (participation rate, 80.1%). We excluded 59 participants who provided indecipherable responses in the open text boxes and 19 participants who completed the survey in less than one-third of the median completion time (2 minutes and 58 seconds), leaving a final analytic sample of 1733 participants. Participants had a mean (SD) age of 41 (15) years (range, 18-87 years); of 1732 with responses available, 1143 identified as female (66.0%), 548 identified as male (31.6%), and 41 provided other gender identity responses (2.4%). Most participants identified as non-Hispanic White (1151 [66.4%]) and most had completed a bachelor’s degree or more (470 [27.1%]). Consistent with sampling quotas, 477 (27.5%) reported having had COVID-19, 499 (28.8%) reported not having had COVID-19 and being at least partially vaccinated, and 509 (29.4%) reported not having had COVID-19 and being unvaccinated. An additional 160 participants (9.2%) were unsure whether they had COVID-19 and were at least partially vaccinated, and 81 (4.7%) were unsure whether they had COVID-19 and were not vaccinated. An additional 7 participants (0.4%) did not respond regarding either their COVID-19 status or their vaccination status. Participant characteristics and additional survey measures are provided in Table 1; the full survey is provided in the eAppendix in the Supplement.

**Table 1.** Participant Characteristics^a^

Characteristic	Values for final sample (N = 1733)
Age, mean (SD) [range], y	41 (15) [18-87]
Age, y	
≥60	241/1724 (14.0)
50-59	229/1724 (13.3)
40-49	325/1724 (18.9)
30-39	481/1724 (27.9)
18-29	448/1724 (26.0)
Gender identity	
Female	1143/1732 (66.0)
Male	548/1732 (31.6)
Prefer not to say	15/1732 (0.9)
Nonbinary/third gender	10/1732 (0.6)
Prefer to self-describe	10/1732 (0.6)
Transgender man	5/1732 (0.3)
Transgender woman	1/1732 (0.1)
Race and ethnicity	
Hispanic	193/1733 (11.1)
Non-Hispanic American Indian or Alaska Native	20/1733 (1.1)
Non-Hispanic Asian or Asian American	45/1733 (2.6)
Non-Hispanic Black or African American	279/1733 (16.1)
Non-Hispanic Native Hawaiian or other Pacific Islander	3/1733 (0.2)
Non-Hispanic White or European American	1151/1733 (66.4)
Non-Hispanic multiracial	26/1733 (1.5)
Non-Hispanic other race	13/1733 (0.7)
Other^b^	3/1733 (0.2)
Educational attainment	
None	16/1731 (0.9)
Elementary school	5/1731 (0.3)
Some high school but no diploma	95/1731 (5.5)
High school (diploma or GED)	606/1731 (35.0)
Some college, but no degree	425/1731 (24.5)
Trade school	114/1731 (6.6)
Bachelor’s degree	354/1731 (20.5)
Master’s degree	95/1731 (5.5)
Doctoral/professional degree	21/1731 (1.2)
Location of residence	
Rural	486/1728 (28.1)
Small city (eg, <100 000 people)	281/1728 (16.3)
Suburb	564/1728 (32.6)
Midsized city (100 000 to 1 million people)	145/1728 (8.4)
Large city (>1 million people)	248/1728 (14.3)
Other	4/1728 (0.2)
Political affiliation	
Democrat	596/1733 (34.4)
Republican	462/1733 (26.7)
Independent	425/1733 (24.5)
Liberal third party	23/1733 (1.3)
Conservative third party	5/1733 (0.3)
No affiliation	222/1733 (12.8)
COVID-19 infection history	
Confident or certain of no COVID-19 infection	1009/1727 (58.4)
Confident or certain of COVID-19 infection	477/1727 (27.5)
Uncertain of COVID-19 infection	241/1727 (14.0)
COVID-19 vaccine status	
Not vaccinated	812/1732 (46.9)
Received BNT162b2 (Pfizer) or mRNA-1273 (Moderna) vaccine	
1 dose	111/1732 (6.4)
2 doses	466/1732 (26.9)
2 doses and a booster	244/1732 (14.1)
Received Ad.26.COV2.S (Johnson & Johnson/Janssen) vaccine	
1 dose	51/1732 (2.9)
1 dose and a booster	48/1732 (2.8)
Source of COVID-19 information^c^	
My physician	1061/1718 (61.7)
Friends	665/1713 (38.8)
Family	809/1713 (47.2)
Local department of health	864/1710 (50.5)
Centers for Disease Control and Prevention	902/1714 (52.6)
Google	730/1716 (42.5)
A certain politician	109/1671 (6.5)
A certain celebrity	85/1680 (5.1)
A certain media personality	111/1674 (6.6)
Survey scores, mean (SD)	
Religiosity^d^	3.96 (2.16)
Political stance on social issues^e^	3.96 (1.66)
Vaccine attitudes^f^	4.67 (2.06)
How often they wear a mask in stores, ^g^	2.88 (1.14)
COVID-19 prevention measures taken more or less relative to others with whom they interact^h^	3.18 (1.22)
Disbelief in science mean (SD)^i^	4.01 (1.50)
Conspiracy theories about COVID-19^j^	2.60 (1.04)
The virus causing COVID-19 was purposefully released by a government or a person	2.94 (1.32)
COVID-19 is a biological weapon being tested	2.80 (1.30)
The current COVID-19 outbreak is a form of population control to reduce the number of people in infected countries	2.73 (1.30)
The COVID-19 vaccine has a microchip so the government can track you	2.38 (1.29)
COVID-19 is not real	2.11 (1.27)
The COVID-19 vaccine causes infertility	2.67 (1.18)

Abbreviation: GED, General Educational Development.

^a^ Unless indicated otherwise, data are expressed as No./total No. (%) of participants. Owing to missing data, numbers of participants may not total 1733 in all categories. Percentages have been rounded and may not total 100.

^b^ Includes participants who reported their race (as White or European American [n = 1] or more than 1 race [n = 2]) but did not report their ethnicity.

^c^ Includes all that apply.

^d^ Scores range from 1 (not at all) to 7 (very).

^e^ Scores range from 1 (very liberal) to 7 (very conservative).

^f^ Scores range from 1 (very negative) to 7 (very positive).

^g^ Scores range from 1 (never) to 4 (every time).

^h^ Scores range from 1 (much less) to 5 (much more).

^i^ Scores range from 1 (strongly agree) to 7 (strongly disagree) for 6 items (Cronbach α = .94).

^j^ Scores range from 1 (definitely false) to 5 (definitely true) for 5 items (Cronbach α = .90).

### Frequency of and Reasons for Misrepresentation and Nonadherence

**Figure 1.**
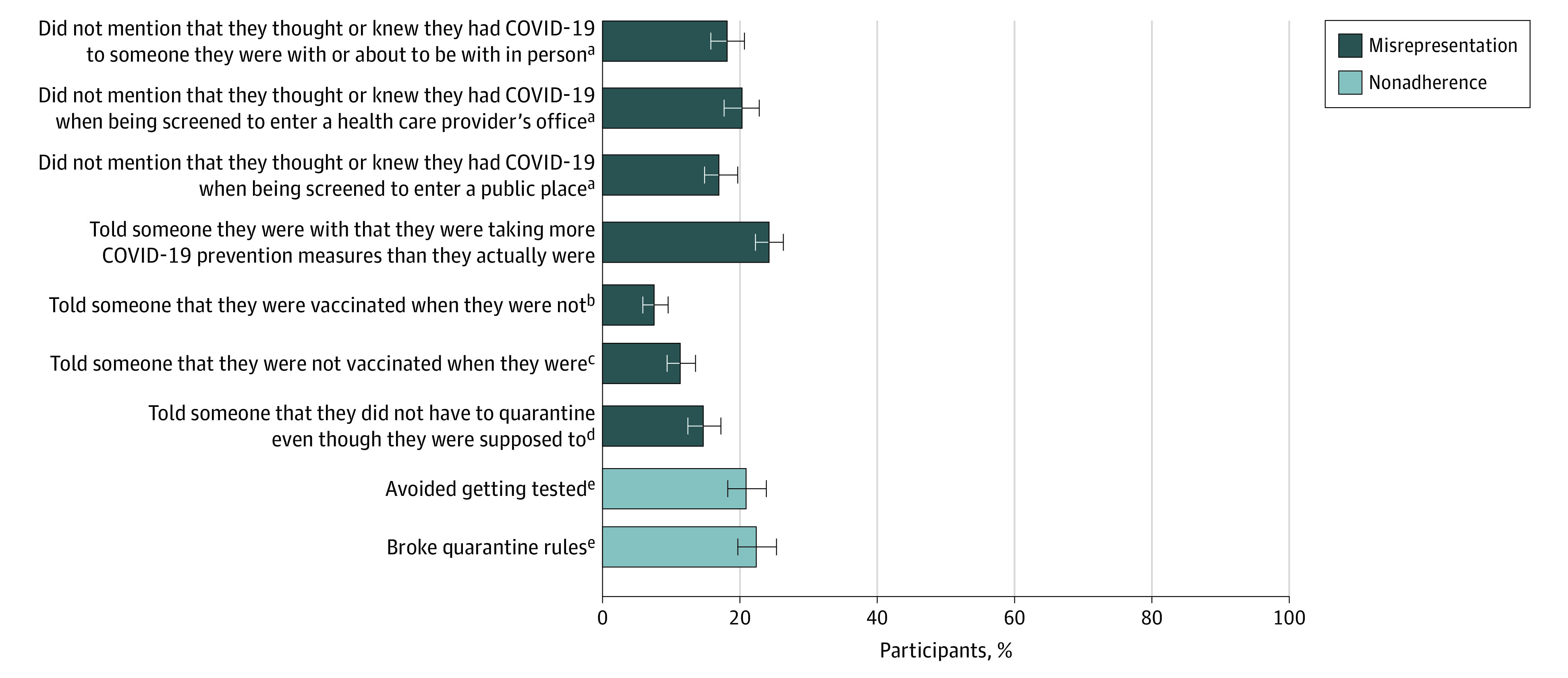
Frequency With Which Participants Engaged in Misrepresentation and Nonadherence Error bars indicate 95% CIs. ^a^Among those who thought or knew they had COVID-19. ^b^Among those who were not vaccinated. ^c^Among those who were vaccinated. ^d^Among those who were told to quarantine. ^e^Among those who thought they had COVID-19.

Overall, 721 participants (41.6%) reported misrepresentation or nonadherence for at least 1 of the 9 behaviors (Figure 1). Among the 9 items, the most commonly reported included telling someone they were with or were about to be with in person that they were taking more COVID-19 preventive measures than they actually were (420 of 1726 [24.3%]), followed by breaking quarantine rules (190 of 845 [22.5%]), avoiding getting tested for COVID-19 when they thought they might have it (171 of 814 [21.0%]), and not mentioning that they thought they might have or knew they had COVID-19 when being screened to enter a clinician’s office (193 of 945 [20.4%]).

Table 2 reports, among those who reported misrepresentation or nonadherence in a given item (eg, broke quarantine rules), the percentage who endorsed each reason offered for this misrepresentation or nonadherence. “I wanted my life to feel ‘normal’ (ie, how I felt before the COVID-19 pandemic began),” “I wanted to exercise my freedom to do what I want,” “It’s no one else’s business,” “I didn’t feel very sick,” and “I was following guidance from a public figure I trust (eg, politicians, scientists, people on the news, celebrities)” were the most commonly endorsed reasons for misrepresentation and nonadherence to COVID-19 public health measures. A substantial proportion of participants also endorsed reasons such as “I didn’t think COVID-19 was real,” “I didn’t think COVID-19 was a big deal,” and “I didn’t want someone to judge or think badly of me.”

**Table 2.** Reasons for Misrepresentation and Nonadherence Regarding COVID-19 Public Health Measures Among Study Participants^a^

Reason	Scenario, No./total No. (%)								
	Did not mention having or possibly having COVID-19			Said they were taking more preventive measures than they were	Said they did not have to quarantine despite requirement to do so	Said they were vaccinated when not vaccinated	Said they were not vaccinated when vaccinated	Did not test when might have COVID-19	Broke quarantine rules
	When screened to enter a clinician’s office	When screened to enter a public place	When meeting or about to meet someone in person						
I wanted my life to feel “normal” (ie, how I felt before the COVID-19 pandemic began)	101/192 (52.6)	94/163 (57.7)	87/171 (50.9)	222/417 (53.2)	66/124 (53.2)	31/62 (50.0)	NA	NA	105/189 (55.5)
I wanted to exercise my freedom to do what I want	91/191 (47.6)	81/163 (49.7)	76/171 (44.4)	190/417 (45.6)	68/125 (54.4)	31/62 (50.0)	NA	79/170 (46.5)	100/189 (52.9)
I was following guidance from a public figure that I trust (eg, politicians, scientists, people on the news, celebrities)	88/190 (46.3)	74/162 (45.7)	75/171 (43.9)	174/415 (41.9)	55/125 (44.0)	22/61 (36.1)	54/104 (51.9)	62/170 (36.5)	70/189 (37.0)
I didn’t feel very sick	84/191 (44.0)	82/163 (50.3)	77/171 (45.0)	NA	70/123 (56.9)	NA	NA	80/170 (47.1)	93/190 (48.9)
It’s no one else’s business	83/191 (43.5)	NA	66/170 (38.8)	167/415 (40.2)	67/126 (53.2)	33/62 (53.2)	57/104 (54.8)	71/169 (42.0)	112/189 (59.3)
I didn’t want to be stopped from doing something I needed to do	81/192 (42.2)	78/162 (48.1)	NA	NA	NA	NA	NA	NA	NA
I didn’t want to have to get tested for COVID-19	80/190 (42.1)	74/161 (46.0)	NA	NA	NA	NA	NA	NA	NA
I didn’t want someone to judge or think badly of me	70/192 (36.5)	64/161 (39.7)	60/172 (34.9)	147/416 (35.3)	48/125 (38.4)	25/62 (40.3)	47/103 (45.6)	54/169 (31.9)	56/189 (29.6)
I didn’t think I really had COVID-19	70/192 (36.5)	71/163 (43.5)	71/171 (41.5)	NA	62/125 (49.6)	NA	NA	63/169 (37.3)	90/189 (47.6)
I didn’t think COVID-19 was a big deal	67/192 (34.9)	67/162 (41.3)	69/171 (40.3)	107/416 (25.7)	51/125 (40.8)	22/62 (35.5)	NA	52/170 (30.6)	75/190 (39.5)
I didn’t want certain people to know	66/191 (34.5)	62/163 (38.0)	56/171 (32.7)	133/416 (32.0)	52/124 (41.9)	25/62 (40.3)	51/104 (49.0)	51/169 (30.2)	59/189 (31.2)
I didn’t think it mattered	64/191 (33.5)	57/162 (35.2)	53/169 (31.4)	117/417 (28.1)	53/125 (42.4)	20/62 (32.3)	55/104 (52.9)	47/169 (27.8)	80/189 (42.3)
I didn’t think COVID-19 was real	60/191 (31.4)	65/163 (39.9)	50/170 (29.4)	104/417 (24.9)	55/123 (44.7)	19/61 (31.1)	NA	50/169 (29.6)	53/188 (28.2)
I was bored or lonely	NA	NA	70/171 (40.9)	139/415 (33.5)	59/125 (47.2)	NA	NA	NA	77/190 (40.5)
I couldn’t miss work to stay home	NA	NA	66/172 (38.4)	140/415 (33.7)	49/125 (39.2)	NA	NA	NA	70/188 (37.2)
I couldn’t miss important nonwork responsibilities to stay home (eg, get groceries, care for loved ones)	NA	NA	75/170 (44.1)	172/415 (41.4)	69/125 (55.2)	NA	NA	NA	NA
I didn’t want them to be angry at me for exposing them	NA	NA	60/169 (35.5)	128/415 (30.8)	51/124 (41.1)	NA	NA	NA	NA
I didn’t want to miss an event or fun activity to stay home	NA	NA	62/171 (36.3)	136/415 (32.8)	52/124 (41.9)	NA	NA	NA	78/189 (41.3)
I was confused about the rules for quarantine	NA	NA	57/170 (33.5)	NA	46/124 (37.1)	NA	NA	NA	61/189 (32.3)
I wanted to be able to do something where being vaccinated was required (eg, a special event, get together with friends/family who were vaccinated, etc)	NA	NA	NA	NA	NA	24/62 (38.7)	NA	NA	NA
I needed to be able to go to work	NA	NA	NA	NA	NA	20/62 (32.3)	NA	NA	NA
I needed to be able to attend college classes	NA	NA	NA	NA	NA	11/62 (17.7)	NA	NA	NA
I didn’t want to have to deal with the consequences of a test showing that I had COVID-19 (eg, my family would have to quarantine, I would have to miss work, my child would miss school, etc)	NA	NA	NA	NA	NA	NA	NA	65/170 (38.2)	NA
I was worried it would hurt or be uncomfortable to get tested	NA	NA	NA	NA	NA	NA	NA	72/170 (42.3)	NA
I wanted to keep COVID-19 rates low in my area so public health measures were not put in place (eg, closing schools, mask mandates)	NA	NA	NA	NA	NA	NA	NA	55/168 (32.7)	NA
I didn’t want the government to have my personal information	NA	NA	NA	NA	NA	NA	NA	61/170 (35.9)	NA
I thought I couldn’t afford the cost of getting tested	NA	NA	NA	NA	NA	NA	NA	51/168 (30.4)	NA
I didn’t have time to get tested	NA	NA	NA	NA	NA	NA	NA	45/168 (26.8)	NA
I didn’t know how or where to get tested	NA	NA	NA	NA	NA	NA	NA	41/170 (24.1)	NA

Abbreviation: NA, not applicable.

^a^ Data are presented as No. (%) of participants.

### Risk Factors Associated With Misrepresentation and Nonadherence

**Figure 2.**
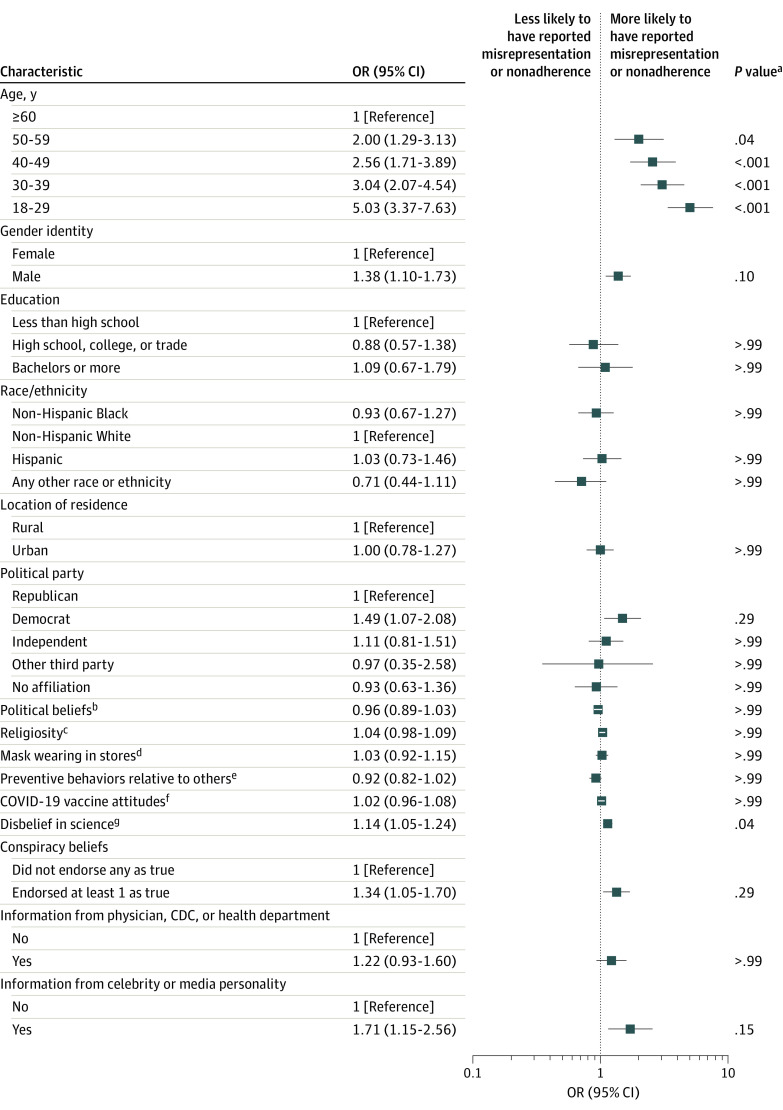
Exploratory Multiple Logistic Regression Analysis of Risk Factors Associated With Misrepresentation and Nonadherence OR indicates odds ratio. ^a^Holm-Bonferroni correction applied. ^b^Mean (SD) score, 3.97 (1.66); scores range from 1 (very liberal) to 7 (very conservative). ^c^Mean (SD) score, 3.96 (2.16); scores range from 1 (not at all) to 7 (very). ^d^Mean (SD) score, 2.88 (1.14); scores range from 1 (never) to 4 (every time). ^e^Mean (SD) score, 3.18 (1.22); scores range from 1 (much less) to 5 (much more). ^f^Mean (SD) score, 4.67 (2.06); scores range from 1 (very negative) to 7 (very positive). ^g^Mean (SD) score, 4.01 (1.50); scores range from 1 (strongly agree) to 7 (strongly disagree).

The exploratory multiple logistic regression^28^ (Tjur *R*^2^ = 0.097; area under the curve, 0.69) indicated that younger participants (odds ratio [OR] for those aged 18-29 years, 4.87 [95% CI, 3.27-7.34]; OR for those aged 30-39 years, 3.16 [95% CI, 2.16-4.70]; OR for those aged 40-49 years, 2.59 [95% CI, 1.73-3.92]; OR for those aged 50-59 years, 2.09 [95% CI, 1.35-3.25]) compared with those 60 years or older and those with greater disbelief in science (OR, 1.14 [95% CI, 1.05-1.23]) had significantly higher odds of reporting misrepresentation or nonadherence in any of the 9 items (Figure 2). Gender identity, educational attainment, race and ethnicity, location of residence, political party affiliation, political beliefs, religiosity, mask use in retail and grocery stores, degree of COVID-19 preventive measures taken relative to others, attitude toward vaccines, COVID-19 conspiracy beliefs, and where COVID-19 information was obtained were not associated with misrepresentation or nonadherence in at least 1 of the 9 items.

## Discussion

The results of this study demonstrate that nearly half of survey participants reported engaging in misrepresentation and/or nonadherence regarding public health measures against COVID-19 during the pandemic. Overstating COVID-19 preventive measures they are taking, breaking quarantine rules, avoiding getting tested for COVID-19 when they thought they might have it, and not mentioning that they thought or knew they had COVID-19 when being screened to enter a clinician’s office were the most common manifestations of misrepresentation and nonadherence. The most common reasons for these behaviors included wanting life to feel normal, wanting to exercise personal freedom, feeling that it is no one else’s business, and not feeling very sick. A substantial minority of participants also explained these behaviors by endorsing statements about COVID-19 not being real or a big deal.

These results are consistent with the little research conducted to date demonstrating misrepresentation and nonadherence regarding COVID-19 public health measures^18,19,20^ and with research demonstrating nondisclosure of non–COVID-19 medical information such as nondisclosure of HIV-positive status to sexual partners^15^ and of important medical information (eg, being depressed or suicidal, recreational drug use) to clincians.^16,17^ Our findings regarding participants’ reasons for misrepresentation and nonadherence (eg, wanting to exercise personal freedom and believing that COVID-19 is not real) are aligned with prior research demonstrating the desire to maintain personal autonomy in response to past governmental health requirements^29,30^ and denials of reputable medical (specifically regarding COVID-19^31,32^ as well as other domains^33,34^) and scientific information.^35,36^

The results also suggest that younger participants and those with greater disbelief in science may be more likely to engage in misrepresentation or nonadherence. Younger patients have been found to have a higher likelihood of dishonesty regarding disclosure of medical information^16,17^ and lower likelihood of medical adherence in general^37,38^ and regarding COVID-19 preventive measures specifically.^39,40^ Similarly, greater disbelief in science has been an important factor associated with nonadherence to health behaviors during the pandemic (eg, masking, vaccination uptake)^41,42^ and beyond.^34,43^ These groups may represent an important focus for efforts to address misrepresentation and nonadherence.

Overall, the results of this study suggest that some participants misrepresented their COVID-19 status and/or vaccination status and did not adhere to public health measures. Although these public health measures can involve tremendous psychological, social, financial, and physical burdens,^6,7,8,9,10,11,12^ such misrepresentation and nonadherence may have placed others at risk of COVID-19. For example, not disclosing thinking or knowing one has COVID-19 when entering a clinician’s office endangers clinicians, office staff, and other patients who may be at increased risk of severe outcomes from COVID-19.

### Limitations

The present research has limitations. First, the online sample is not fully representative of the US population, although it allowed for a larger and more diverse sample size than data collected using other means and ensured the inclusion of sufficient participants differing in COVID-19 history and vaccination status. We also acknowledge that with an online nonprobability sample, the findings from our model do not offer unbiased population level insights and should be interpreted with caution. These findings provide important preliminary insights for hypothesis testing about factors that may be associated with misrepresentation or nonadherence regarding COVID-19 public health guidelines. Second, in a study examining the extent to which people are misrepresenting the truth, participants may have been dishonest in their survey responses. However, if this occurred, participants likely provided answers that were more socially desirable^44^ (ie, minimizing their misrepresentation or nonadherence), thereby likely making our results an underestimate of how commonly people misrepresent or are nonadherent in this setting. Third, although the study captured the percentage of individuals who misrepresented or were nonadherent, it cannot speak to the frequency or the degree of these behaviors. In addition, indicated reasons for misrepresentation and nonadherence may have been post hoc explanations, rather than the original determinants. Finally, there may have been other reasons why people misrepresented or were nonadherent that we did not measure (eg, prosocial or practical reasons). For instance, participants may have avoided getting a COVID-19 test because they did not want to risk infecting others at the testing site. In addition, participants may not have mentioned a COVID-19 diagnosis or symptoms in a screening because they thought they were no longer contagious and therefore believed it to be irrelevant. This could have led us to overestimate the cases of misrepresentation and nonadherence that are of concern (ie, misrepresentation and nonadherence that may lead to increased spread of the disease). However, screening questions typically include a time frame that preempts this possibility (eg, “Have you had any symptoms of COVID-19 in the last 2 weeks?”). Furthermore, the general public is often uninformed about viral transmission^45^; therefore, full disclosure is necessary for the medical screener to assess actual risk level.

## Conclusions

The results of this survey study reveal a serious public health challenge for the COVID-19 pandemic and any future infectious disease outbreaks. With misrepresentation and nonadherence regarding public health measures being fairly common, the effectiveness of these measures in preventing disease spread may be undermined. This possibility highlights the need for further research examining strategies for educating the public on the importance of honesty and adherence in these situations as well as for addressing the factors (eg, the burden of quarantine, believing that COVID-19 is not real) that drive misrepresentation and nonadherence. It also underscores the importance of public health officials, policy makers, and media personalities fostering trust and engagement in these public health measures to reduce the occurrence and therefore the impact of misrepresentation and nonadherence.

## Notes

Supplement. **eMethods.** Survey Development Information **eTable.** Individual Regression Models **eAppendix.** Full Study Survey
