# Supplementary material for: Misrepresentation and Nonadherence Regarding COVID-19 Public Health Measures
Source: JAMA Netw Open. 2022 Oct 10;5(10):e2235837. doi: 10.1001/jamanetworkopen.2022.35837 (PMC9552890; doi:10.1001/jamanetworkopen.2022.35837)
Supplement: Supplement. — eMethods. Survey Development Information eTable. Individual Regression Models eAppendix. Full Study Survey [file jamanetwopen-e2235837-s001.pdf]

Supplementary Online Content

Levy AG, Thorpe A, Scherer LD, et al. Misrepresentation and nonadherence regarding COVID-19 public health measures. *JAMA Netw Open*. 2022;5(10):e2235837. doi:10.1001/jamanetworkopen.2022.35837

eMethods. Survey Development Information
eTable. Individual Regression Models
eAppendix. Full Study Survey

This supplementary material has been provided by the authors to give readers additional information about their work.

© 2022 Levy AG et al. *JAMA Network Open*.

eMethods. Survey Development Information

Throughout the development of the survey, members of the study team sought feedback from a convenience sample including undergraduate students. These individuals were asked to complete the survey multiple times to report on its comprehensibility (e.g., did they understand the questions) and mechanics (e.g., technical errors in the coding of the survey), as well as any omissions or redundancies in the survey.

We had several mechanisms in place through Qualtrics to prevent the survey from being completed by bots/survey screening programs. First, we prevented multiple submissions so participants could not take the survey multiple times. Second, we used Google's invisible reCaptcha as a bot detection technology; all participants included in the final sample had a reCaptcha score greater than .6 (A score of greater than or equal to 0.5 means the participants is likely a human. A score of less than 0.5 means the participants is likely a bot.). Third, we also enabled security scan monitor which prevents email security scanning software from accidently starting surveys. Fourth, we blocked search engines from including our survey in their search results limiting access only to those who had been invited.

© 2022 Levy AG et al. *JAMA Network Open*.

eTable. Individual Regression Models

		Original model (all 9 questions)		7 misrepresentation questions		2 non-adherence questions	
		OR	*p **	*OR*	*p **	*OR*	*p **
Age (ref: 60+)							
	50-59	2.00 (1.29, 3.13)	**.044**	2.16 (1.34, 3.53)	.036	1.57 (0.85, 2.95)	1.00
	40-49	2.56 (1.71, 3.89)	**<.001**	2.91 (1.88, 4.60)	**<.001**	2.39 (1.40, 4.25)	**.044**
	30-39	3.04 (2.07, 4.54)	**<.001**	3.29 (2.16, 5.10)	**<.001**	2.12 (1.26, 3.72)	.136
	18-29	5.03 (3.37, 7.63)	**<.001**	5.96 (3.88, 9.38)	**<.001**	3.04 (1.79, 5.37)	**.002**
Gender identity (ref: Female)							
	Male	1.38 (1.10, 1.73)	.104	1.29 (1.02, 1.63)	.550	1.26 (0.95, 1.67)	1.00
Education (ref: less than High school)							
	High School, College, or Trade	0.88 (0.57, 1.38)	1.00	0.82 (0.52, 1.31)	1.00	1.18 (0.68, 2.14)	1.00
	Bachelors or more	1.09 (0.67, 1.79)	1.00	1.10 (0.67, 1.84)	1.00	1.26 (0.69, 2.42)	1.00
Race/Ethnicity (ref: Non-Hispanic White)							
	Non-Hispanic black	0.93 (0.67, 1.27)	1.00	0.91 (0.65, 1.26)	1.00	0.88 (0.59, 1.32)	1.00
	Hispanic	1.03 (0.73, 1.46)	1.00	0.95 (0.67, 1.35)	1.00	1.38 (0.91, 2.04)	1.00
	Any other race/ethnicity	0.71 (0.44, 1.11)	1.00	0.74 (0.45, 1.17)	1.00	0.61 (0.30, 1.11)	1.00
Residence (Ref: Rural)							
	Urban	1.00 (0.78, 1.27)	1.00	0.96 (0.74, 1.23)	1.00	1.14 (0.84, 1.56)	1.00
Political party (Ref: Republican)							
	Democrat	1.49 (1.07, 2.08)	.285	1.56 (1.11, 2.19)	.190	0.90 (0.60, 1.34)	1.00
	Independent	1.11 (0.81, 1.51)	1.00	1.07 (0.77, 1.47)	1.00	0.84 (0.57, 1.23)	1.00
	Other third Party	0.97 (0.35, 2.58)	1.00	0.72 (0.24, 1.97)	1.00	1.19 (0.36, 3.47)	1.00
	No political party affiliation	0.93 (0.63, 1.36)	1.00	0.78 (0.52, 1.18)	1.00	0.95 (0.59, 1.50)	1.00
Political beliefs		0.96 (0.89, 1.03)	1.00	0.95 (0.88, 1.03)	1.00	0.94 (0.85, 1.03)	1.00
Religiosity		1.04 (0.98, 1.09)	1.00	1.02 (0.97, 1.08)	1.00	1.06 (0.99, 1.13)	1.00
Mask wearing in stores		1.03 (0.92, 1.15)	1.00	1.13 (1.00, 1.28)	.631	0.87 (0.76, 1.00)	1.00
Preventative behaviors relative to others		0.92 (0.82, 1.02)	1.00	0.90 (0.81, 1.01)	1.00	0.89 (0.78, 1.01)	1.00
COVID-19 vaccine attitudes		1.02 (0.96, 1.08)	1.00	1.04 (0.98, 1.11)	1.00	1.02 (0.95, 1.11)	1.00
Disbelief in science		1.14 (1.05, 1.24)	**.044**	1.12 (1.03, 1.22)	.191	1.15 (1.03, 1.27)	.221
Conspiracy beliefs (ref: Did not endorse any as true)							
	Endorsed ≥1 as true	1.34 (1.05, 1.70)	.285	1.40 (1.09, 1.79)	.155	1.35 (1.00, 1.83)	.987
Info from Dr/CDC/Health Dept (ref: No)							
	Yes	1.22 (0.93, 1.60)	1.00	1.19 (0.90, 1.59)	1.00	1.03 (0.74, 1.45)	1.00
Info from Celeb/Media personality (ref: No)							
	Yes	1.71 (1.15, 2.56)	.145	1.87 (1.26, 2.79)	**.038**	1.32 (0.84, 2.04)	1.00

© 2022 Levy AG et al. *JAMA Network Open*.

Observations/ R^2 Tjur	*1612/0.097*	*1612/0.109*	*1612/0.053*

© 2022 Levy AG et al. *JAMA Network Open*.

eAppendix. Full Study Survey

Start of Block: Introduction

consent **Description of research by the investigator:** The purpose of this research study is to learn more about people's experiences and attitudes towards COVID-19 and the COVID-19 vaccines, and about the impact that COVID-19 has had on our interactions with others.

Participation time: Participating in this study involves taking part in a single survey, which will take 5-10 minutes.

Risks: Any risks to you associated with this study are not expected to be greater than anything you encounter in your everyday life. Some of the questions may cause stress or emotional discomfort. You do not have to answer any questions you do not want to. All data collected for this study will be stored on a password-protected computer. The study team will not have access to your name or other identifying information. If there were a security breach to the study database, an unauthorized person could access your answers but not your name. This could result in a loss of privacy. The risk of this occurring is very low but is a possibility, nonetheless.

Benefits: You may not experience any direct benefit from being in this study. However, sharing your insight and experience will benefit care and wellbeing during this trying time and during other potential pandemics.

Conflict of interest: There are no conflicts of interest to report.

Confidentiality: This survey is being conducted online using the Qualtrics survey panel and the data will be stored and analyzed at the University of Utah and Middlesex Community College. The survey is optional and completely anonymous. No identifying information will be required.

Person to contact: If you have any questions, complaints or if you feel you have been harmed by this research please contact Angela Fagerlin, PhD, Professor and Chair of the Department of Population Health Sciences. 801-587-0049. Contact the Institutional Review Board (IRB) if you have questions regarding your rights as a research participant. Also, contact the IRB if you have questions, complaints or concerns which you do not feel you can discuss with the investigator. The University of Utah IRB may be reached by phone at (801) 581-3655 or by e-

© 2022 Levy AG et al. *JAMA Network Open*.

mail at irb@hsc.utah.edu.

Costs to participants and compensation: Qualtrics will pay participants for completing each survey. It should take less than 20 minutes to complete the questionnaire. Participation in this study is voluntary. You can choose not to take part. You can choose not to answer any question you prefer not to answer, but you will only be paid for the survey if you submit it.

By completing this questionnaire, you are giving your consent to participate.

We appreciate your willingness to share your experience during the COVID-19 pandemic.

Page Break

© 2022 Levy AG et al. *JAMA Network Open*.

intro Welcome!

In this survey, you will answer some questions about your experiences and opinions regarding the COVID-19 pandemic. We are interested in your personal experiences and opinions.

Click the arrow button to begin.

End of Block: Introduction

Start of Block: Screener Qs

X→

HadCoV Do you think you have had COVID-19?

- ◯ No: I'm pretty sure or certain that I have not had COVID-19 (1)
- ◯ Yes: I'm pretty sure or certain that I have had COVID-19 (2)
- ◯ I am not sure if I have had COVID-19 (3)

Display This Question:

If HadCoV = 2

X→

HadCoV_How How did you conclude that you had COVID-19? (If you have had COVID-19 more than once, mark all that apply).

- ▢ I had a COVID-19 test which said that I had COVID-19 (I had a positive COVID-19 test) (1)
- ▢ I did not have a COVID-19 test, but health care provider told me I probably had COVID-19 (2)
- ▢ I did not have COVID-19 test, but I assumed I had COVID-19 based on my symptoms and/or exposure to COVID-19 (3)

Page Break

© 2022 Levy AG et al. *JAMA Network Open*.

VaxStatus Which of the following describes your COVID-19 vaccination status?

- I have not received a COVID-19 vaccine (1)
- I have received one dose of either a Pfizer or Moderna vaccine (2)
- I have received two doses of a either a Pfizer or Moderna vaccine (3)
- I have received two doses of a either a Pfizer or Moderna vaccine AND a booster (4)
- I have received one dose of the Johnson & Johnson vaccine (5)
- I have received one dose of the Johnson & Johnson vaccine AND a booster (6)

Display This Question:

If VaxStatus = 3

Or VaxStatus = 5

VaxBoost How likely are you to get a COVID-19 vaccine booster shot?

- I will definitely get a booster (1)
- I will probably get a booster (2)
- I don’t know if I will get a booster (3)
- I will probably not get a booster (4)
- I will definitely not get a booster (5)

Page Break

© 2022 Levy AG et al. *JAMA Network Open*.

Display This Question:

If HadCoV = 2

And If

VaxStatus = 2

Or VaxStatus = 3

Or VaxStatus = 4

Or VaxStatus = 5

Or VaxStatus = 6

WhenHadCoV When did you get COVID-19?

- ◯ Before I got a COVID-19 vaccine (1)
- ◯ After I got a COVID-19 vaccine (2)
- ◯ Both before and after I got a COVID-19 vaccine (3)

End of Block: Screener Qs

Start of Block: Prior Exposure and Vaccines

Display This Question:

If VaxStatus = 1

VaxIntent How likely are you to get a COVID-19 vaccine?

- ◯ I will definitely not get a vaccine (1)
- ◯ I will probably not get a vaccine (2)
- ◯ I don't know if I will get a vaccine (3)
- ◯ I will probably get a vaccine (4)
- ◯ I will definitely get a vaccine (5)

Page Break

© 2022 Levy AG et al. *JAMA Network Open*.

VaxBnftHadCoV As far as you know, what is the benefit of a COVID-19 vaccine to a person who has already had COVID-19?

- ○ No benefit at all (1)
- ○ A small benefit (2)
- ○ A moderate benefit (3)
- ○ A lot of benefit (4)
- ○ Not sure (5)

VaxSafeHadCoV As far as you know, how safe is a COVID-19 vaccine for a person who has already had COVID-19?

- ○ Very unsafe (1)
- ○ Unsafe (2)
- ○ Safe (3)
- ○ Very safe (4)
- ○ Not sure (5)

© 2022 Levy AG et al. *JAMA Network Open*.

VaxBnftNoCoV As far as you know, what is the benefit of a COVID-19 vaccine to a person who has not had COVID-19?

- ○ No benefit at all (1)
- ○ A small benefit (2)
- ○ A moderate benefit (3)
- ○ A lot of benefit (4)
- ○ Not sure (5)

VaxSafeNoCoV As far as you know, how safe is a COVID-19 vaccine for a person who has not had COVID-19?

- ○ Very unsafe (1)
- ○ Unsafe (2)
- ○ Safe (3)
- ○ Very safe (4)
- ○ Not sure (5)

End of Block: Prior Exposure and Vaccines

Start of Block: Lying General

LyingTxt On the following pages, we will ask you about several situations in which some people avoid sharing information. For each situation, we will ask you whether you have ever avoided sharing the information and if so, the reasons why. We just want to better understand how people are thinking and making decisions during COVID-19. Please remember that this survey does not ask for your name or any other identifying information. Thank you again for your help.

© 2022 Levy AG et al. *JAMA Network Open*.

GenLie_Disagree Have you ever avoided telling a health care provider that you disagreed with his/her recommendation for you?

- No (1)
- Yes (2)

GenLie_Exer Have you ever avoided telling a health care provider that you did not exercise, or did not exercise regularly?

- No (1)
- Yes (2)

GenLie_Depres Have you ever avoided telling a health care provider that you were depressed or how depressed you were?

- No (1)
- Yes (2)

GenLie_Drug Have you ever avoided telling a health care provider that you used recreational drugs or how much you used recreational drugs?

- No (1)
- Yes (2)

End of Block: Lying General

Start of Block: Lying CoV

© 2022 Levy AG et al. *JAMA Network Open*.

CoVSymp During the pandemic, did you ever have symptoms that you thought might be COVID-19?

- ○ No (1)
- ○ Yes (2)

Page Break

© 2022 Levy AG et al. *JAMA Network Open*.

Display This Question:

If CoVSymp != 1

Or HadCoV != 1

CoVLie_PrvdrScreen **You said that during the pandemic, you thought you might have COVID-19 (or you knew you had it) maybe because of a positive test or symptoms you were having.**

Did you ever not mention that you thought you might have COVID-19 (or you knew you had it) when being screened to enter **a health care provider's office** (e.g., doctor, nurse, dentist, therapist)?

- No (1)
- Yes (2)

Display This Choice:

If CoVSymp != 2

And HadCoV != 2

- I never thought I had or might have COVID-19 (3)

Display This Question:

If CoVSymp != 1

Or HadCoV != 1

CoVLie_PublicScreen Did you ever not mention that you thought you might have COVID-19 (or you knew you had it) when being screened to enter **a public place** (e.g., grocery store, restaurant, gym, going on an airplane, etc.)?

- No (1)
- Yes (2)

Display This Choice:

If HadCoV != 2

And CoVSymp != 2

- I never thought I had or might have COVID-19 (3)

Display This Question:

If CoVSymp != 1

Or HadCoV != 1

CoVLie_InPerson Did you ever not mention that you thought you might have COVID-19 (or you knew you had it) to **someone you were with, or were about to see in-person**?

- ○ No (1)
- ○ Yes (2)

Display This Choice:

If HadCoV != 2

And CoVSymp != 2

- ○ I never thought I had or might have COVID-19 (3)

Page Break

© 2022 Levy AG et al. *JAMA Network Open*.

CoVLie_Precau Have you ever told someone that you were with, or were about to see in-person, that you were taking more measures to prevent COVID-19 than you actually were? For example, have you ever said that you were always wearing protective masks indoors even though you weren't or that you hadn't been with people outside of your "bubble" or "pod" even though you had?

- No (1)
- Yes (2)

Page Break

© 2022 Levy AG et al. *JAMA Network Open*.

Display This Question:

If VaxStatus = 1

CoVLie_HadVax Have you ever told someone that you were vaccinated for COVID-19 even though you were not vaccinated?

- ○ No (1)
- ○ Yes (2)

Display This Question:

If VaxStatus = 1

CoVLie_Exmpt Have you ever said that you have a medical or religious reason for not getting a COVID-19 vaccine <u>even though you didn't really have such a reason</u>?

- ○ No (1)
- ○ Yes (2)

Display This Question:

If VaxStatus = 2

Or VaxStatus = 3

Or VaxStatus = 4

Or VaxStatus = 5

Or VaxStatus = 6

CoVLie_NoVax Have you ever told someone that you were not vaccinated for COVID-19 even though you were vaccinated?

- ○ No (1)
- ○ Yes (2)

Display This Question:

If CoVSymp != 1

© 2022 Levy AG et al. *JAMA Network Open*.

CoVLie_Tested **You said that during the pandemic, you thought you might have COVID-19 because of symptoms you were having.**

Have you ever avoided getting tested for COVID-19 when you thought you might have it?

- No (1)
- Yes (2)

Display This Choice:

If CoVSymp != 2

- I never thought I might have COVID-19 (3)

Page Break

© 2022 Levy AG et al. *JAMA Network Open*.

QteenRules_Txt **During the COVID-19 pandemic, there have been quarantine rules for those who test positive for COVID-19, have symptoms of COVID-19, or who have been exposed to someone with COVID-19. The quarantine rules are that the person must stay home at all times and not be around people outside of their household at all.**

AskedToQteen During the COVID-19 pandemic, have you ever been told to follow quarantine rules?

- ○ No (1)
- ○ Yes (2)

Page Break

© 2022 Levy AG et al. *JAMA Network Open*.

Display This Question:

If AskedToQteen != 1

CoVLie_Qteen Have you ever told someone that you didn't need to quarantine even though you were supposed to?

- ◯ No (1)
- ◯ Yes (2)

Display This Choice:

If AskedToQteen != 2

- ◯ I have never been told to follow quarantine rules (3)

Display This Question:

If AskedToQteen != 1

CoVBrk_Qteen Have you ever <u>broken quarantine rules</u> (e.g., left the house or were around people outside of your household during your quarantine period)?

- ◯ No (1)
- ◯ Yes (2)

Display This Choice:

If AskedToQteen != 2

- ◯ I have never been told to follow quarantine rules (3)

Page Break

© 2022 Levy AG et al. *JAMA Network Open*.

ChildU18 Are you a parent or step-parent of any children under the age of 18?

- No (1)
- Yes (2)

Skip To: End of Block If ChildU18 = 1

Page Break

© 2022 Levy AG et al. *JAMA Network Open*.

ChildU18Lived Have any of your children or step-children under the age of 18 lived with you during the pandemic?

- ◯ No (1)
- ◯ Yes (2)

Display This Choice:

If ChildU18 != 2

- ◯ I am not a parent or step-parent of any children under the age of 18 (3)

Skip To: End of Block If ChildU18Lived != 2

Page Break

© 2022 Levy AG et al. *JAMA Network Open*.

ChildCoVSymp **Please answer the following questions about any of your children or step-children under the age of 18 who have lived with you during the pandemic.**

During the pandemic, did any of your children ever have symptoms that you thought might be COVID-19?

- ○ No (1)
- ○ Yes (2)

HadCoV_kid
Do you think any of your children have had COVID-19?

- ○ No: I'm pretty sure or certain that none of my children have had COVID-19 (1)
- ○ Yes: I'm pretty sure or certain that at least one of my children have had COVID-19 (2)
- ○ Yes: I'm pretty sure or certain that all of my children have had COVID-19 (3)
- ○ I am not sure if any of my children have had COVID-19 (4)

Page Break

© 2022 Levy AG et al. *JAMA Network Open*.

Display This Question:

If ChildCoVSymp != 1

Or HadCoV_kid != 1

And ChildCoVSymp , 1 Is Displayed

CoVLie_InPerson_kid **Please answer the following questions about any of your children or step-children under the age of 18 who have lived with you during the pandemic.**

You said that during the pandemic, you thought at least one of your children might have COVID-19 (or you knew they had it) either because of a positive test or symptoms they were having.

Did you ever not mention that you thought your child might have COVID-19 (or you knew they had it) to someone your child was with, or was about to see in-person?

- ○ No (1)
- ○ Yes (2)

Display This Choice:

If ChildCoVSymp != 2

And If

HadCoV_kid != 2

And HadCoV_kid != 3

- ○ I never thought my child had or might have COVID-19 (3)

Display This Question:

If ChildCoVSymp != 1

And ChildCoVSymp , 1 Is Displayed

CoVLie_Tested_kid **Please answer the following questions about any of your children or step-children under the age of 18 who have lived with you during the pandemic.**

You said that during the pandemic, you thought at least one of your children might have COVID-19 because of symptoms they were having.

© 2022 Levy AG et al. *JAMA Network Open*.

Have you ever avoided getting your child tested for COVID-19 when you thought they might have it?

○ No (1)

○ Yes (2)

Display This Choice:

If ChildCoVSymp != 2

○ I never thought my child might have COVID-19 (3)

Page Break

© 2022 Levy AG et al. *JAMA Network Open*.

QteenRules_Txt **During the COVID-19 pandemic, there have been quarantine rules for those who test positive for COVID-19, have symptoms of COVID-19, or who have been exposed to someone with COVID-19. The quarantine rules are that the person must stay home at all times and not be around people outside of their household at all.**

AskedToQteen_kid **Please answer the following questions about any of your children or step-children under the age of 18 who have lived with you during the pandemic.**

During the COVID-19 pandemic, have any of your children ever been told to follow quarantine rules?

- ○ No (1)
- ○ Yes (2)

Page Break

© 2022 Levy AG et al. *JAMA Network Open*.

Display This Question:

If AskedToQteen_kid , 2 Is Displayed

And AskedToQteen_kid != 1

CoVLie_Qteen_kid **Please answer the following questions about any of your children or step-children under the age of 18 who have lived with you during the pandemic.**

You said that during the pandemic, at least one of your children was told to follow quarantine rules.

Have you ever told someone that your child didn't need to quarantine even though they were supposed to?

- No (1)
- Yes (2)

Display This Choice:

If AskedToQteen_kid != 2

- My child has never been told to follow quarantine rules (3)

Display This Question:

If AskedToQteen_kid , 2 Is Displayed

And AskedToQteen_kid != 1

CoVBrk_Qteen_kid
Please answer the following questions about any of your children or step-children under the age of 18 who have lived with you during the pandemic.
Have you ever allowed your child to break quarantine rules (e.g., left the house or were around people outside of your household during their quarantine period)?

- No (1)
- Yes (2)

Display This Choice:

If AskedToQteen_kid != 2

- My child has never been told to follow quarantine rules (3)

© 2022 Levy AG et al. *JAMA Network Open*.

Page Break

© 2022 Levy AG et al. *JAMA Network Open*.

CoVLie_kidAge **Please answer the following questions about any of your children or step-children under the age of 18 who have lived with you during the pandemic.**

Have you ever said that your child was older than they actually were so that they could get a COVID-19 vaccine?

○ No (1)

○ Yes (2)

Display This Question:

If CoVLie_kidAge = 1

CoVLie_kidLook **Please answer the following questions about any of your children or step-children under the age of 18 who have lived with you during the pandemic.**

Do you have any children that you think you could have passed off as being old enough for the COVID-19 vaccine (even though you didn't try to do so)?

○ No (1)

○ Yes (2)

Page Break

© 2022 Levy AG et al. *JAMA Network Open*.

ChildVaxed **Please answer the following questions about any of your children or step-children under the age of 18 who have lived with you during the pandemic.**

For any of your children who are old enough to get a COVID-19 vaccine, have they received a vaccine?

- ○ No: None of my children are old enough to get a vaccine (1)
- ○ No: None of my children who are old enough to get a COVID-19 vaccine have gotten a vaccine (2)
- ○ Yes: Some, but not all, of my children who are old enough to get a COVID-19 vaccine have gotten a vaccine (3)
- ○ Yes: All my children who are old enough to get a COVID-19 vaccine have gotten a vaccine (4)

Page Break

© 2022 Levy AG et al. *JAMA Network Open*.

Display This Question:

If ChildVaxed = 2

Or ChildVaxed = 3

Or ChildVaxed = 1

CoVLie_HadVax_kid
Please answer the following questions about any of your children or step-children under the age of 18 who have lived with you during the pandemic.

Have you ever told someone that your child was vaccinated for COVID-19 even though they were not vaccinated?

○ No (1)

○ Yes (2)

Display This Question:

If ChildVaxed = 4

Or ChildVaxed = 3

CoVLie_NoVax_kid **Please answer the following questions about any of your children or step-children under the age of 18 who have lived with you during the pandemic.**

Have you ever told someone that your child was not vaccinated for COVID-19 even though they were vaccinated?

○ No (1)

○ Yes (2)

End of Block: Lying CoV

Start of Block: Lying CoV reasons

© 2022 Levy AG et al. *JAMA Network Open*.

Display This Question:

If CoVLie_PrvdrScreen = 2

CoVLie_Prvdr_rzn Please think back to the time or times that you did not mention that you thought you might have COVID-19 (or you knew you had it) when being screened to enter a

© 2022 Levy AG et al. *JAMA Network Open*.

health care provider's office (e.g., doctor, nurse, dentist, therapist). <u>There are many reasons why people might do this. Why did you?</u>

© 2022 Levy AG et al. *JAMA Network Open*.

	Yes (2)	No (1)
I didn’t think I really had COVID-19. (1)	○	○
I didn't feel very sick. (2)	○	○
I didn't want someone to judge or think badly of me. (3)	○	○
I didn’t think it mattered. (4)	○	○
It's no one else's business. (5)	○	○
I didn’t think COVID-19 was real. (6)	○	○
I didn’t think COVID-19 was a big deal. (7)	○	○
I was following guidance from a public figure that I trust (e.g., politicians, scientists, people on the news, celebrities). (8)	○	○
I didn't want certain people to know. (9)	○	○
I wanted to exercise my freedom to do what I want. (10)	○	○
I didn’t want to have to get tested for COVID-19. (11)	○	○
I didn’t want to be stopped from doing something I needed to do. (i.e., have my appointment with the health care provider). (13)	○	○
I wanted my life to feel “normal” (i.e., how I felt before the COVID-19 pandemic began). (14)	○	○

© 2022 Levy AG et al. *JAMA Network Open*.

Display This Question:

If CoVLie_PrvdrScreen = 2

CoVLie_Prvdr_rznTxt Are there any other reasons why you did not mention that you thought you might have COVID-19 (or you knew you had it) when being screened to enter a health care provider's office (e.g., doctor, nurse, dentist)? Also use the box below if you have any other comments you would like to make about your responses.

____

____

____

____

____

Display This Question:

If CoVLie_Prvdr_rzn = 3 [2]

CoVLie_Pvdr_judge You said that one reason why you did not mention that you thought you might have COVID-19 (or you knew you had it) when being screened to enter a health care provider's office (e.g., doctor, nurse, dentist, therapist) was because you thought someone might judge or think badly of you. Why did you think someone would judge or think badly of you?

____

____

____

____

____

Page Break

© 2022 Levy AG et al. *JAMA Network Open*.

Display This Question:

If CoVLie_PublicScreen = 2

CoVLie_Public_rzn Please think back to the time or times that you did not mention that you thought you might have COVID-19 (or you knew you had it) when being screened to enter a

© 2022 Levy AG et al. *JAMA Network Open*.

public place (e.g., grocery store, restaurant, gym, going on an airplane, etc). There are many reasons why people might do this. Why did you?

© 2022 Levy AG et al. *JAMA Network Open*.

	Yes (2)	No (1)
I didn't think I really had COVID-19. (CoVLie_Public_rzn_1)	○	○
I didn't feel very sick. (CoVLie_Public_rzn_2)	○	○
I didn't want someone to judge or think badly of me. (CoVLie_Public_rzn_3)	○	○
I didn't think it mattered. (CoVLie_Public_rzn_4)	○	○
I didn't think COVID-19 was real. (CoVLie_Public_rzn_5)	○	○
I didn't think COVID-19 was a big deal. (CoVLie_Public_rzn_6)	○	○
I was following guidance from a public figure that I trust (e.g., politicians, scientists, people on the news, celebrities). (CoVLie_Public_rzn_7)	○	○
I didn't want certain people to know. (CoVLie_Public_rzn_8)	○	○
I wanted to exercise my freedom to do what I want. (CoVLie_Public_rzn_9)	○	○
I didn't want to have to get tested for COVID-19. (CoVLie_Public_rzn_10)	○	○
I didn't want to be stopped from doing something I needed to do (e.g., go to the grocery store). (CoVLie_Public_rzn_11)	○	○
I wanted my life to feel "normal" (i.e., how I felt before the COVID-19 pandemic began). (CoVLie_Public_rzn_12)	○	○

© 2022 Levy AG et al. *JAMA Network Open*.

Display This Question:

If CoVLie_PublicScreen = 2

CoVLie_Public_rznTxt Are there any other reasons why you did not mention that you thought you might have COVID-19 (or you knew you had it) when being screened to enter a public place (e.g., grocery store, restaurant, gym, going on an airplane, etc)? Also use the box below if you have any other comments you would like to make about your responses.

____

____

____

____

____

Display This Question:

If CoVLie_Public_rzn = 3 [2]

CoVLie_Public_judge You said that one reason why you did not mention that you thought you might have COVID-19 (or you knew you had it) when being screened to enter a public place (e.g., grocery store, restaurant, gym, going on an airplane, etc) was because you thought someone might judge or think badly of you. Why did you think someone would judge or think badly of you?

____

____

____

____

____

Page Break

© 2022 Levy AG et al. *JAMA Network Open*.

Display This Question:

If CoVLie_InPerson = 2

© 2022 Levy AG et al. *JAMA Network Open*.

CoVLie_inPers_rzn Please think back to the time or times that you did not mention that you thought you might have COVID-19 (or you knew you had it) to someone you were with, or were about to see in-person. There are many reasons why people might do this. Why did you?

© 2022 Levy AG et al. *JAMA Network Open*.

	Yes (2)	No (1)
I didn’t think I really had COVID-19. (1)	○	○
I didn’t feel very sick. (2)	○	○
I didn’t want someone to judge or think badly of me. (3)	○	○
I didn’t think it mattered. (4)	○	○
It’s no one else’s business. (5)	○	○
I didn’t think COVID-19 was real. (6)	○	○
I didn’t think COVID-19 was a big deal. (7)	○	○
I was following guidance from a public figure that I trust (e.g., politicians, scientists, people on the news, celebrities). (8)	○	○
I didn’t want certain people to know. (9)	○	○
I wanted to exercise my freedom to do what I want. (10)	○	○
I didn’t want them to be angry at me for exposing them. (11)	○	○
I was confused about the rules for quarantine. (12)	○	○
I was bored or lonely. (13)	○	○
I wanted my life to feel “normal” (i.e., how I felt before the COVID-19 pandemic began). (14)	○	○

© 2022 Levy AG et al. *JAMA Network Open*.

I couldn't miss work to stay home. (15)	○	○
I couldn't miss important non-work responsibilities to stay home (e.g., get groceries, care for loved ones). (16)	○	○
I didn't want to miss an event or other fun activity to stay home. (17)	○	○

Display This Question:

If CoVLie_InPerson = 2

CoVLie_inPers_rznTxt Are there any other reasons why you did not mention that you thought you might have COVID-19 (or you knew you had it) to someone you were with, or were about to see in-person. Also use the box below if you have any other comments you would like to make about your responses.

____

____

____

____

____

Display This Question:

If CoVLie_inPers_rzn = 3 [2]

CoVLie_inPers_judge You said that one reason why you did not mention that you thought you might have COVID-19 (or you knew you had it) to someone you were with, or were about to see in-person was because you thought someone might judge or think badly of you. Why did you think someone would judge or think badly of you?

____

____

____

© 2022 Levy AG et al. *JAMA Network Open*.

Page Break

© 2022 Levy AG et al. *JAMA Network Open*.

Display This Question:

If CoVLie_Precau = 2

© 2022 Levy AG et al. *JAMA Network Open*.

CoVLie_Precau_rzn Please think back to the time or times that you told someone that you were with, or were about to see in-person, that you were taking more measures to prevent COVID-19 than you actually were. There are many reasons why people might do this. Why did you?

© 2022 Levy AG et al. *JAMA Network Open*.

	Yes (2)	No (1)
I didn’t want someone to judge or think badly of me. (1)	○	○
I didn’t think it mattered. (2)	○	○
It’s no one else’s business. (3)	○	○
I didn’t think COVID-19 was real. (4)	○	○
I didn’t think COVID-19 was a big deal. (5)	○	○
I was following guidance from a public figure that I trust (e.g., politicians, scientists, people on the news, celebrities). (6)	○	○
I wanted to exercise my freedom to do what I want. (7)	○	○
I didn’t want them to be angry at me for exposing them. (8)	○	○
I was bored or lonely. (9)	○	○
I didn’t want certain people to know. (10)	○	○
I wanted my life to feel “normal” (i.e., how I felt before the COVID-19 pandemic began). (11)	○	○
I couldn’t miss work to stay home. (12)	○	○
I couldn’t miss important non-work responsibilities to stay home (e.g., get groceries, care for loved ones). (13)	○	○

© 2022 Levy AG et al. *JAMA Network Open*.

I didn't want to miss an event or other fun activity to stay home. (14)	○	○

Display This Question:

If CoVLie_Precau = 2

CoVLie_Precau_rznTxt Are there any other reasons why you told someone that you were with, or were about to see in-person, that you were taking more measures to prevent COVID-19 than you actually were? Also use the box below if you have any other comments you would like to make about your responses.

____

____

____

____

____

Display This Question:

If CoVLie_Precau_rzn = 1 [2]

CoVLie_Precau_judge You said that one reason why you told someone that you were with, or were about to see in-person, that you were taking more measures to prevent COVID-19 than you actually were was because you thought someone might judge or think badly of you. Why did you think someone would judge or think badly of you?

____

____

____

____

____

© 2022 Levy AG et al. *JAMA Network Open*.

Page Break

© 2022 Levy AG et al. *JAMA Network Open*.

Display This Question:

If CoVLie_HadVax = 2

© 2022 Levy AG et al. *JAMA Network Open*.

CoVLie_HadVax_rzn Please think back to the time or times that you told someone that you were vaccinated for COVID-19 even though you were not vaccinated. There are many reasons why people might do this. Why did you?

© 2022 Levy AG et al. *JAMA Network Open*.

	Yes (2)	No (1)
I didn't think it mattered. (1)	○	○
I didn't want someone to judge or think badly of me. (2)	○	○
I wanted to be able to do something where being vaccinated was required (e.g., go to a special event, get together with friends or family who were vaccinated, etc). (3)	○	○
I wanted to exercise my freedom to do what I want. (4)	○	○
I was following guidance from a public figure that I trust (e.g., politicians, scientists, people on the news, celebrities). (5)	○	○
I didn't think COVID-19 was real. (6)	○	○
I needed to be able to go to work. (7)	○	○
I needed to be able to attend college classes. (8)	○	○
I didn't think COVID-19 was a big deal. (9)	○	○
I wanted my life to feel "normal" (i.e., how I felt before the COVID-19 pandemic began). (10)	○	○
It's no one else's business. (11)	○	○
I didn't want certain people to know. (12)	○	○

© 2022 Levy AG et al. *JAMA Network Open*.

Display This Question:

If CoVLie_HadVax = 2

CoVLie_HadVax_rznTxt Are there any other reasons why you told someone that you were vaccinated for COVID-19 even though you were not vaccinated? Also use the box below if you have any other comments you would like to make about your responses.

____

____

____

____

____

Display This Question:

If CoVLie_HadVax_rzn = 2 [2]

CoVLie_HadVax_judge You said that one reason why you told someone that you were vaccinated for COVID-19 even though you were not vaccinated was because you thought someone might judge or think badly of you. Why did you think someone would judge or think badly of you?

____

____

____

____

____

Page Break

© 2022 Levy AG et al. *JAMA Network Open*.

Display This Question:

If CoVLie_Exmpt = 2

© 2022 Levy AG et al. *JAMA Network Open*.

CoVLie_VaxExe_rzn Please think back to the time or times that you said that you have a medical or religious reason for not getting a COVID-19 vaccine even though you didn’t really have such a reason. There are many reasons why people might do this. Why did you?

© 2022 Levy AG et al. *JAMA Network Open*.

	Yes (2)	No (1)
I didn't think it mattered. (1)	○	○
I didn't want someone to judge or think badly of me. (2)	○	○
I wanted to exercise my freedom to do what I want. (3)	○	○
I was following guidance from a public figure that I trust (e.g., politicians, scientists, people on the news, celebrities). (4)	○	○
I wanted to be able to do something where being vaccinated was required (e.g., go to a special event, get together with friends or family who were vaccinated, etc). (5)	○	○
I needed to be able to go to work. (6)	○	○
I needed to be able to attend college classes. (7)	○	○
I didn't think COVID-19 was real. (8)	○	○
I didn't think COVID-19 was a big deal. (9)	○	○
I wanted my life to feel "normal" (i.e., how I felt before the COVID-19 pandemic began). (10)	○	○
It's no one else's business. (11)	○	○
I didn't want certain people to know. (12)	○	○

© 2022 Levy AG et al. *JAMA Network Open*.

Display This Question:

If CoVLie_Exmpt = 2

CoVLie_VaxExe_rznTxt Are there any other reasons why you said that you had a medical or religious reason for not getting a COVID-19 vaccine even though you didn't really have such a reason? Also use the box below if you have any other comments you would like to make about your responses.

____

____

____

____

____

Display This Question:

If CoVLie_VaxExe_rzn = 2 [2]

CoVLie_VaxExe_judge You said that one reason why you said that you had a medical or religious reason for not getting a COVID-19 vaccine even though you didn't really have such a reason was because you thought someone might judge or think badly of you. Why did you think someone would judge or think badly of you?

____

____

____

____

____

Page Break

© 2022 Levy AG et al. *JAMA Network Open*.

Display This Question:

If CoVLie_NoVax = 2

CoVLie_NoVax_rzn Please think back to the time or times that you told someone that you were not vaccinated for COVID-19 even though you were vaccinated. There are many reasons why people might do this. Why did you?

	Yes (2)	No (1)
I didn't think it mattered. (1)	○	○
I didn't want someone to judge or think badly of me. (2)	○	○
I was following guidance from a public figure that I trust (e.g., politicians, scientists, people on the news, celebrities). (3)	○	○
It's no one else's business. (4)	○	○
I didn't want certain people to know. (5)	○	○

Display This Question:

If CoVLie_NoVax = 2

CoVLie_NoVax_rznTxt Are there any other reasons why you told someone that you were not vaccinated for COVID-19 even though you were vaccinated? Also use the box below if you have any other comments you would like to make about your responses.

____

____

____

____

____

© 2022 Levy AG et al. *JAMA Network Open*.

Display This Question:

If CoVLie_NoVax_rzn = 2 [2]

CoVLie_NoVax_judge You said that one reason why you told someone that you were not vaccinated for COVID-19 even though you were vaccinated was because you thought someone might judge or think badly of you. Why did you think someone would judge or think badly of you?

____

____

____

____

____

Page Break

© 2022 Levy AG et al. *JAMA Network Open*.

Display This Question:

If CoVLie_Tested = 2

© 2022 Levy AG et al. *JAMA Network Open*.

CoVLie_Tested_rzn Please think back to the time or times that you avoided getting tested for COVID-19 when you thought you might have it. There are many reasons why people might do this. Why did you?

© 2022 Levy AG et al. *JAMA Network Open*.

	Yes (2)	No (1)
I didn’t think I really had COVID-19. (CoVLie_Tested_rzn_1)	○	○
I didn’t feel very sick. (CoVLie_Tested_rzn_2)	○	○
I didn’t want someone to judge or think badly of me. (CoVLie_Tested_rzn_3)	○	○
I didn’t think it mattered. (CoVLie_Tested_rzn_4)	○	○
It’s no one else’s business. (CoVLie_Tested_rzn_5)	○	○
I didn’t think COVID-19 was real. (CoVLie_Tested_rzn_6)	○	○
I didn’t think COVID-19 was a big deal. (CoVLie_Tested_rzn_7)	○	○
I was following guidance from a public figure that I trust (e.g., politicians, scientists, people on the news, celebrities). (CoVLie_Tested_rzn_8)	○	○
I wanted to exercise my freedom to do what I want. (CoVLie_Tested_rzn_9)	○	○
I didn’t want certain people to know. (CoVLie_Tested_rzn_10)	○	○
I didn’t know how or where to get tested. (CoVLie_Tested_rzn_11)	○	○
I was worried it would hurt or be uncomfortable to get tested. (CoVLie_Tested_rzn_12)	○	○
I thought I couldn’t afford the cost of getting tested. (CoVLie_Tested_rzn_13)	○	○

© 2022 Levy AG et al. *JAMA Network Open*.

I didn’t have time to get tested. (CoVLie_Tested_rzn_14)	◯	◯
I didn’t want the government to have my personal medical information. (CoVLie_Tested_rzn_15)	◯	◯
I wanted to keep COVID-19 rates low in my area so public health measures were not put in place (e.g., closing schools, mask mandates). (CoVLie_Tested_rzn_16)	◯	◯
I didn’t want to have to deal with the consequences of a test showing that I had COVID-19 (e.g., my family would have to quarantine, I would have to miss work, my child would miss school, etc). (CoVLie_Tested_rzn_17)	◯	◯

Display This Question:

If CoVLie_Tested = 2

CoVLie_Tested_rznTxt Are there any other reasons why you avoided getting tested for COVID-19 when you thought you might have it? Also use the box below if you have any other comments you would like to make about your responses.

____

____

____

____

____

© 2022 Levy AG et al. *JAMA Network Open*.

Display This Question:

If CoVLie_Tested_rzn = 20 [2]

CoVLie_Tested_judge You said that one reason why you avoided getting tested for COVID-19 when you thought you might have it was because you thought someone might judge or think badly of you. Why did you think someone would judge or think badly of you?

____

____

____

____

____

Page Break

© 2022 Levy AG et al. *JAMA Network Open*.

Display This Question:

If CoVLie_Qteen = 2

© 2022 Levy AG et al. *JAMA Network Open*.

CoVLie_Qteen_rzn Please think back to the time or times that you told someone that you didn't need to quarantine even though you were supposed to. There are many reasons why people might do this. Why did you?

© 2022 Levy AG et al. *JAMA Network Open*.

	Yes (2)	No (1)
I didn't think I really had COVID-19. (CoVLie_Qteen_rzn_1)	○	○
I didn't feel very sick. (CoVLie_Qteen_rzn_2)	○	○
I didn't want someone to judge or think badly of me. (CoVLie_Qteen_rzn_3)	○	○
I didn't think it mattered. (CoVLie_Qteen_rzn_4)	○	○
I didn't think COVID-19 was real. (CoVLie_Qteen_rzn_5)	○	○
I didn't think COVID-19 was a big deal. (CoVLie_Qteen_rzn_6)	○	○
I was following guidance from a public figure that I trust (e.g., politicians, scientists, people on the news, celebrities). (CoVLie_Qteen_rzn_7)	○	○
It's no one else's business. (CoVLie_Qteen_rzn_8)	○	○
I didn't want certain people to know. (CoVLie_Qteen_rzn_9)	○	○
I wanted to exercise my freedom to do what I want. (CoVLie_Qteen_rzn_10)	○	○
I was confused about the rules for quarantine. (CoVLie_Qteen_rzn_11)	○	○
I was bored or lonely. (CoVLie_Qteen_rzn_12)	○	○
I wanted my life to feel "normal" (i.e., how I felt before the COVID-19 pandemic began). (CoVLie_Qteen_rzn_13)	○	○

© 2022 Levy AG et al. *JAMA Network Open*.

I couldn’t miss work to stay home. (CoVLie_Qteen_rzn_14)	○	○
I couldn’t miss important non-work responsibilities to stay home (e.g., get groceries, care for loved ones). (CoVLie_Qteen_rzn_15)	○	○
I didn’t want to miss an event or other fun activity to stay home. (CoVLie_Qteen_rzn_16)	○	○
I didn't want them to be angry at me for exposing them. (CoVLie_Qteen_rzn_17)	○	○

Display This Question:

If CoVLie_Qteen = 2

CoVLie_Qteen_txt Are there any other reasons why you told someone that you didn't need to quarantine even though you were supposed to? Also use the box below if you have any other comments you would like to make about your responses.

____

____

____

____

____

Display This Question:

If CoVLie_Qteen_rzn = 19 [2]

CoVLie_Qteen_jdg You said that one reason why you told someone that you didn't need to quarantine even though you were supposed to was because you thought someone might judge or think badly of you. Why did you think someone would judge or think badly of you?

____

© 2022 Levy AG et al. *JAMA Network Open*.

Page Break

© 2022 Levy AG et al. *JAMA Network Open*.

Display This Question:

If CoVBrk_Qteen = 2

© 2022 Levy AG et al. *JAMA Network Open*.

CoVBrk_Qteen_rzn Please think back to the time or times that you broke quarantine rules. There are many reasons why people might do this. Why did you?

© 2022 Levy AG et al. *JAMA Network Open*.

	Yes (2)	No (1)
I didn't think I really had COVID-19. (CoVBrk_Qteen_rzn_1)	○	○
I didn't feel very sick. (CoVBrk_Qteen_rzn_2)	○	○
I didn't want someone to judge or think badly of me. (CoVBrk_Qteen_rzn_3)	○	○
I didn't think it mattered. (CoVBrk_Qteen_rzn_4)	○	○
I didn't think COVID-19 was real. (CoVBrk_Qteen_rzn_5)	○	○
I didn't think COVID-19 was a big deal. (CoVBrk_Qteen_rzn_6)	○	○
I was following guidance from a public figure that I trust (e.g., politicians, scientists, people on the news, celebrities). (CoVBrk_Qteen_rzn_7)	○	○
It's no one else's business. (CoVBrk_Qteen_rzn_8)	○	○
I didn't want certain people to know. (CoVBrk_Qteen_rzn_9)	○	○
I wanted to exercise my freedom to do what I want. (CoVBrk_Qteen_rzn_10)	○	○
I was confused about the rules for quarantine. (CoVBrk_Qteen_rzn_11)	○	○
I was bored or lonely. (CoVBrk_Qteen_rzn_12)	○	○
I wanted my life to feel "normal" (i.e., how I felt before the COVID-19 pandemic began). (CoVBrk_Qteen_rzn_13)	○	○

© 2022 Levy AG et al. *JAMA Network Open*.

I couldn't miss work to stay home. (CoVBrk_Qteen_rzn_14)	○	○
I couldn't miss important non-work responsibilities to stay home (e.g., get groceries, care for loved ones). (CoVBrk_Qteen_rzn_15)	○	○
I didn't want to miss an event or other fun activity to stay home. (CoVBrk_Qteen_rzn_16)	○	○

Display This Question:

If CoVBrk_Qteen = 2

CoVBrk_Qteen_rzn Are there any other reasons why you broke quarantine rules? Also use the box below if you have any other comments you would like to make about your responses.

____

____

____

____

____

Display This Question:

If CoVBrk_Qteen_rzn = 19 [2]

CoVBrk_Qteen_judge You said that one reason why you broke quarantine rules was because you thought someone might judge or think badly of you. Why did you think someone would judge or think badly of you?

____

____

____

© 2022 Levy AG et al. *JAMA Network Open*.

Page Break

© 2022 Levy AG et al. *JAMA Network Open*.

Display This Question:

If CoVLie_InPerson_kid = 2

CoVLie_inPers_rznKid **Please answer the following questions about any of your children or step-children under the age of 18 who have lived with you during the pandemic.**

Please think back to the time or times that you did not mention that you thought your child

© 2022 Levy AG et al. *JAMA Network Open*.

might have COVID-19 (or you knew they had it) to someone your child was with, or was about to see in-person. <u>There are many reasons why people might do this. Why did you?</u>

© 2022 Levy AG et al. *JAMA Network Open*.

	Yes (2)	No (1)
I didn’t think my child really had COVID-19. (CoVLie_inPers_rznKid_1)	○	○
My child didn’t feel very sick. (CoVLie_inPers_rznKid_2)	○	○
I didn’t want someone to judge or think badly of me or my child. (CoVLie_inPers_rznKid_3)	○	○
I didn’t think it mattered. (CoVLie_inPers_rznKid_4)	○	○
It’s no one else’s business. (CoVLie_inPers_rznKid_5)	○	○
I didn’t think COVID-19 was real. (CoVLie_inPers_rznKid_6)	○	○
I didn’t think COVID-19 was a big deal. (CoVLie_inPers_rznKid_7)	○	○
I was following guidance from a public figure that I trust (e.g., politicians, scientists, people on the news, celebrities). (CoVLie_inPers_rznKid_8)	○	○
I didn’t want certain people to know. (CoVLie_inPers_rznKid_9)	○	○
I wanted to exercise my freedom to do what I want with my child. (CoVLie_inPers_rznKid_10)	○	○
I didn’t want them to be angry at me or my child for exposing them. (CoVLie_inPers_rznKid_11)	○	○
I was confused about the rules for quarantine. (CoVLie_inPers_rznKid_12)	○	○

© 2022 Levy AG et al. *JAMA Network Open*.

My child was bored or lonely. (CoVLie_inPers_rznKid_13)	○	○
I wanted my child's life to feel "normal" (i.e., how they felt before the COVID-19 pandemic began). (CoVLie_inPers_rznKid_14)	○	○
I didn't want my child to miss school to stay home. (CoVLie_inPers_rznKid_15)	○	○
I didn't want my child to miss important activities to stay home (e.g., music, sports, clubs). (CoVLie_inPers_rznKid_16)	○	○
I didn't want my child to miss an event or other fun activity to stay home. (CoVLie_inPers_rznKid_17)	○	○
I couldn't miss work to stay home. (CoVLie_inPers_rznKid_18)	○	○
I couldn't miss important non-work responsibilities to stay home (e.g., get groceries, care for loved ones). (CoVLie_inPers_rznKid_19)	○	○
I didn't want to miss an event or other fun activity to stay home. (CoVLie_inPers_rznKid_20)	○	○

Display This Question:

If CoVLie_InPerson_kid = 2

CoVLie_inPers_TxtKid Are there any other reasons why you did not mention that you thought your child might have COVID-19 (or you knew they had it) to someone your child was with, or

© 2022 Levy AG et al. *JAMA Network Open*.

was about to see in-person? Also use the box below if you have any other comments you would like to make about your responses.

____

____

____

____

____

Display This Question:

If CoVLie_inPers_rznKid = 22 [2]

CoVLie_inPers_JdgKid You said that one reason why you did not mention that you thought your child might have COVID-19 (or you knew they had it) to someone your child was with, or was about to see in-person was because you thought someone might judge or think badly of you or your child. Why did you think someone would judge or think badly of you or your child?

____

____

____

____

____

Page Break

© 2022 Levy AG et al. *JAMA Network Open*.

Display This Question:

If CoVLie_Tested_kid = 2

CoVLie_Tested_RznKid **Please answer the following questions about any of your children or step-children under the age of 18 who have lived with you during the pandemic.**

Please think back to the time or times that you avoided getting your child tested for COVID-19

© 2022 Levy AG et al. *JAMA Network Open*.

when you thought they might have it. There are many reasons why people might do this. Why did you?

© 2022 Levy AG et al. *JAMA Network Open*.

	Yes (2)	No (1)
I didn’t think my child really had COVID-19. (CoVLie_Tested_RznKid_1)	○	○
My child didn’t feel very sick. (CoVLie_Tested_RznKid_2)	○	○
I didn’t want someone to judge or think badly of me or my child. (CoVLie_Tested_RznKid_3)	○	○
I didn’t think it mattered. (CoVLie_Tested_RznKid_4)	○	○
It’s no one else’s business. (CoVLie_Tested_RznKid_5)	○	○
I didn’t think COVID-19 was real. (CoVLie_Tested_RznKid_6)	○	○
I didn’t think COVID-19 was a big deal. (CoVLie_Tested_RznKid_7)	○	○
I was following guidance from a public figure that I trust (e.g., politicians, scientists, people on the news, celebrities). (CoVLie_Tested_RznKid_8)	○	○
I didn’t want certain people to know. (CoVLie_Tested_RznKid_9)	○	○
I wanted to exercise my freedom to do what I want with my child. (CoVLie_Tested_RznKid_10)	○	○
I wanted to keep COVID-19 rates low in my area so public health measures were not put in place (e.g., closing schools, mask mandates). (CoVLie_Tested_RznKid_11)	○	○

© 2022 Levy AG et al. *JAMA Network Open*.

I didn't know how or where to get my child tested. (CoVLie_Tested_RznKid_12)	◯	◯
My child and/or I were worried it would hurt or be uncomfortable to get tested. (CoVLie_Tested_RznKid_13)	◯	◯
I thought I couldn't afford the cost of getting my child tested. (CoVLie_Tested_RznKid_14)	◯	◯
I didn't have time to get my child tested. (CoVLie_Tested_RznKid_15)	◯	◯
I didn't want the government to have my child's personal medical information. (CoVLie_Tested_RznKid_16)	◯	◯
I didn't want to have to deal with the consequences of a test showing that my child had COVID-19 (e.g., my family would have to quarantine, I would have to miss work, my child would miss school, etc). (CoVLie_Tested_RznKid_17)	◯	◯

Display This Question:

If CoVLie_Tested_kid = 2

CoVLie_Tested_TxtKid Are there any other reasons why you avoided getting your child tested for COVID-19 when you thought that they might have it? Also use the box below if you have any other comments you would like to make about your responses.

____

____

____

____

© 2022 Levy AG et al. *JAMA Network Open*.

____

Display This Question:

If CoVLie_Tested_RznKid = 20 [2]

CoVLie_Tested_JdgKid You said that one reason why you avoided getting your child tested for COVID-19 when you thought that they might have it was because you thought someone might judge or think badly of you or your child. <u>Why did you think someone would judge or think badly of you or your child?</u>

____

____

____

____

____

Page Break

© 2022 Levy AG et al. *JAMA Network Open*.

Display This Question:

If CoVLie_Qteen_kid = 2

CoVLie_Qteen_kid_rzn **Please answer the following questions about any of your children or step-children under the age of 18 who have lived with you during the pandemic.**

Please think back to the time or times that you told someone that your child didn't need to

© 2022 Levy AG et al. *JAMA Network Open*.

quarantine even though they were supposed to. There are many reasons why people might do this. Why did you?

© 2022 Levy AG et al. *JAMA Network Open*.

	Yes (2)	No (1)
I didn’t think my child really had COVID-19. (CoVLie_Qteen_kid_rzn_1)	○	○
My child didn’t feel very sick. (CoVLie_Qteen_kid_rzn_2)	○	○
I didn’t want someone to judge or think badly of me or my child. (CoVLie_Qteen_kid_rzn_3)	○	○
I didn’t think it mattered. (CoVLie_Qteen_kid_rzn_4)	○	○
I didn’t think COVID-19 was real. (CoVLie_Qteen_kid_rzn_5)	○	○
I didn’t think COVID-19 was a big deal. (CoVLie_Qteen_kid_rzn_6)	○	○
I was following guidance from a public figure that I trust (e.g., politicians, scientists, people on the news, celebrities). (CoVLie_Qteen_kid_rzn_7)	○	○
It’s no one else’s business. (CoVLie_Qteen_kid_rzn_8)	○	○
I didn’t want certain people to know. (CoVLie_Qteen_kid_rzn_9)	○	○
I wanted to exercise my freedom to do what I want with my child. (CoVLie_Qteen_kid_rzn_10)	○	○
I was confused about the rules for quarantine. (CoVLie_Qteen_kid_rzn_11)	○	○
My child was bored or lonely. (CoVLie_Qteen_kid_rzn_12)	○	○

© 2022 Levy AG et al. *JAMA Network Open*.

I wanted my child's life to feel "normal" (i.e., how they felt before the COVID-19 pandemic began). (CoVLie_Qteen_kid_rzn_13)	○	○
I didn't want my child to miss school to stay home. (CoVLie_Qteen_kid_rzn_14)	○	○
I didn't want my child to miss important activities to stay home (e.g., music, sports, clubs). (CoVLie_Qteen_kid_rzn_15)	○	○
I didn't want my child to miss an event or other fun activity to stay home. (CoVLie_Qteen_kid_rzn_16)	○	○
I couldn't miss work to stay home. (CoVLie_Qteen_kid_rzn_17)	○	○
I couldn't miss important non-work responsibilities to stay home (e.g., get groceries, care for loved ones). (CoVLie_Qteen_kid_rzn_18)	○	○
I didn't want to miss an event or other fun activity to stay home. (CoVLie_Qteen_kid_rzn_19)	○	○
I didn't want them to be angry at me or my child for exposing them. (CoVLie_Qteen_kid_rzn_20)	○	○

Display This Question:

If CoVLie_Qteen_kid = 2

© 2022 Levy AG et al. *JAMA Network Open*.

CoVLie_Qteen_kidTxt Are there any other reasons why you told someone that your child didn't need to quarantine even though they were supposed to? Also use the box below if you have any other comments you would like to make about your responses.

____

____

____

____

____

Display This Question:

If CoVLie_Qteen_kid_rzn = 24 [2]

CoVLie_Qteen_kidJdge You said that one reason why you told someone that your child didn't need to quarantine even though they were supposed to was because you thought someone might judge or think badly of you. Why did you think someone would judge or think badly of you or your child?

____

____

____

____

____

Page Break

© 2022 Levy AG et al. *JAMA Network Open*.

Display This Question:

If CoVBrk_Qteen_kid = 2

CoVBrk_Qteen_RznKid **Please answer the following questions about any of your children or step-children under the age of 18 who have lived with you during the pandemic.**

© 2022 Levy AG et al. *JAMA Network Open*.

Please think back to the time or times that you allowed your child to break quarantine rules. <u>There are many reasons why people might do this. Why did you?</u>

© 2022 Levy AG et al. *JAMA Network Open*.

	Yes (2)	No (1)
I didn't think my child really had COVID-19. (CoVBrk_Qteen_RznKid_1)	○	○
My child didn't feel very sick. (CoVBrk_Qteen_RznKid_2)	○	○
I didn't want someone to judge or think badly of me or my child. (CoVBrk_Qteen_RznKid_3)	○	○
I didn't think it mattered. (CoVBrk_Qteen_RznKid_4)	○	○
I didn't think COVID-19 was real. (CoVBrk_Qteen_RznKid_5)	○	○
I didn't think COVID-19 was a big deal. (CoVBrk_Qteen_RznKid_6)	○	○
I was following guidance from a public figure that I trust (e.g., politicians, scientists, people on the news, celebrities). (CoVBrk_Qteen_RznKid_7)	○	○
It's no one else's business. (CoVBrk_Qteen_RznKid_8)	○	○
I didn't want certain people to know. (CoVBrk_Qteen_RznKid_9)	○	○
I wanted to exercise my freedom to do what I want with my child. (CoVBrk_Qteen_RznKid_10)	○	○
I was confused about the rules for quarantine. (CoVBrk_Qteen_RznKid_11)	○	○
My child was bored or lonely. (CoVBrk_Qteen_RznKid_12)	○	○

© 2022 Levy AG et al. *JAMA Network Open*.

I wanted my child's life to feel "normal" (i.e., how they felt before the COVID-19 pandemic began). (CoVBrk_Qteen_RznKid_13)	○	○
I didn't want my child to miss school to stay home. (CoVBrk_Qteen_RznKid_14)	○	○
I didn't want my child to miss important activities to stay home (e.g., music, sports, clubs). (CoVBrk_Qteen_RznKid_15)	○	○
I didn't want my child to miss an event or other fun activity to stay home. (CoVBrk_Qteen_RznKid_16)	○	○
I couldn't miss work to stay home. (CoVBrk_Qteen_RznKid_17)	○	○
I couldn't miss important non-work responsibilities to stay home (e.g., get groceries, care for loved ones). (CoVBrk_Qteen_RznKid_18)	○	○
I didn't want to miss an event or other fun activity to stay home. (CoVBrk_Qteen_RznKid_19)	○	○

Display This Question:

If CoVBrk_Qteen_kid = 2

CoVBrk_Qteen_TxtKid Are there any other reasons why you allowed your child to break quarantine rules? Also use the box below if you have any other comments you would like to make about your responses.

____

____

____

Display This Question:

If CoVBrk_Qteen_RznKid = 24 [2]

CoVBrk_Qteen_JdgKid You said that one reason why you allowed your child to break quarantine rules was because you thought someone might judge or think badly of you or your child. Why did you think someone would judge or think badly of you or your child?

Page Break

© 2022 Levy AG et al. *JAMA Network Open*.

Display This Question:

If CoVLie_kidAge = 2

CoVLie_Older_RznKid **Please answer the following questions about any of your children or step-children under the age of 18 who have lived with you during the pandemic.**

Please think back to the time or times that you said that your child was older than they actually were so that they could get a COVID-19 vaccine. There are many reasons why people might do this. Why did you?

	Yes (2)	No (1)
I wanted my child vaccinated to lower their risk of COVID-19 in time for an event or activity that they were participating in. (CoVLie_Older_RznKid_1)	○	○
I wanted my child vaccinated to lower their risk of COVID-19 in time for school or camp. (CoVLie_Older_RznKid_2)	○	○
I wanted my child vaccinated to lower their risk of COVID-19 in time for a trip or a visit with family or friends. (CoVLie_Older_RznKid_3)	○	○
I wanted my child vaccinated to lower their risk of COVID-19 in general. (CoVLie_Older_RznKid_4)	○	○
I wanted to exercise my freedom to do what I want with my child. (CoVLie_Older_RznKid_5)	○	○

Display This Question:

If CoVLie_kidAge = 2

© 2022 Levy AG et al. *JAMA Network Open*.

CoVLie_Older_TxtKid Are there any other reasons why you said that your child was older than they actually were so that they could get a COVID-19 vaccine? Also use the box below if you have any other comments you would like to make about your responses.

____

____

____

____

____

Page Break

© 2022 Levy AG et al. *JAMA Network Open*.

Display This Question:

If CoVLie_HadVax_kid = 2

CoVLie_HadVax_RznKid **Please answer the following questions about any of your children or step-children under the age of 18 who have lived with you during the pandemic.**

Please think back to the time or times that you told someone that your child was vaccinated for

© 2022 Levy AG et al. *JAMA Network Open*.

COVID-19 even though they were not vaccinated. There are many reasons why people might do this. Why did you?

	Yes (2)	No (1)
I didn’t think it mattered. (CoVLie_HadVax_RznKid_1)	○	○
I didn’t want someone to judge or think badly of me or my child. (CoVLie_HadVax_RznKid_2)	○	○
I wanted my child’s life to feel “normal” (i.e., how they felt before the COVID-19 pandemic began). (CoVLie_HadVax_RznKid_3)	○	○
I was following guidance from a public figure that I trust (e.g., politicians, scientists, people on the news, celebrities). (CoVLie_HadVax_RznKid_4)	○	○
It’s no one else’s business. (CoVLie_HadVax_RznKid_5)	○	○
I wanted to exercise my freedom to do what I want with my child. (CoVLie_HadVax_RznKid_6)	○	○
I didn’t think COVID-19 was real. (CoVLie_HadVax_RznKid_7)	○	○
I didn’t think COVID-19 was a big deal. (CoVLie_HadVax_RznKid_8)	○	○
I didn’t want certain people to know. (CoVLie_HadVax_RznKid_24)	○	○
I wanted my child to be able to do something where being vaccinated was required (e.g., go to a special event, get together with friends or family who were vaccinated, etc). (CoVLie_HadVax_RznKid_25)	○	○

© 2022 Levy AG et al. *JAMA Network Open*.

Display This Question:

If CoVLie_HadVax_kid = 2

CoVLie_HadVax_TxtKid Are there any other reasons why you told someone that your child was vaccinated for COVID-19 even though they were not vaccinated? Also use the box below if you have any other comments you would like to make about your responses.

____

____

____

____

____

Display This Question:

If CoVLie_HadVax_RznKid = 17 [2]

CoVLie_HadVax_JdgKid You said that one reason why you told someone that your child was vaccinated for COVID-19 even though they were not vaccinated was because you thought someone might judge or think badly of you or your child. Why did you think someone would judge or think badly of you or your child?

____

____

____

____

____

Page Break

© 2022 Levy AG et al. *JAMA Network Open*.

Display This Question:

If CoVLie_NoVax_kid = 2

CoVLie_NoVax_RznKid **Please answer the following questions about any of your children or step-children under the age of 18 who have lived with you during the pandemic.**

Please think back to the time or times that you told someone that your child was not vaccinated for COVID-19 even though they were vaccinated. There are many reasons why people might do this. Why did you?

	Yes (2)	No (1)
I didn't think it mattered. (CoVLie_NoVax_RznKid_1)	○	○
I didn't want someone to judge or think badly of me or my child. (CoVLie_NoVax_RznKid_2)	○	○
I was following guidance from a public figure that I trust (e.g., politicians, scientists, people on the news, celebrities). (CoVLie_NoVax_RznKid_3)	○	○
It's no one else's business. (CoVLie_NoVax_RznKid_4)	○	○
I didn't want certain people to know. (CoVLie_NoVax_RznKid_5)	○	○

Display This Question:

If CoVLie_NoVax_kid = 2

CoVLie_NoVax_TxtKid Are there any other reasons why you told someone that your child was not vaccinated for COVID-19 even though they were vaccinated? Also use the box below if you have any other comments you would like to make about your responses.

____

____

____

© 2022 Levy AG et al. *JAMA Network Open*.

Display This Question:

If CoVLie_NoVax_RznKid = 11 [2]

CoVLie_NoVax_JdgKid You said that one reason why you told someone that your child was not vaccinated for COVID-19 even though they were vaccinated was because you thought someone might judge or think badly of you or your child. <u>Why did you think someone would judge or think badly of you or your child?</u>

____

____

____

____

____

End of Block: Lying CoV reasons

Start of Block: Vax and Masking

Display This Question:

If VaxIntent = 1

Or VaxIntent = 2

Or VaxIntent = 3

AnonVaxIntent Some people have reported secretly getting a COVID-19 vaccine (not telling anyone or going to a different town so none of their friends or neighbors would know). Would you be more likely to get a COVID-19 vaccine if you could do so without anyone knowing?

- No (1)
- Yes (2)
- Maybe (3)

© 2022 Levy AG et al. *JAMA Network Open*.

Display This Question:

If VaxStatus != 1

AnonVax Some people have reported secretly getting a COVID-19 vaccine (not telling anyone or going to a different town so none of their friends or neighbors would know). Did you receive the vaccine secretly (i.e., did not talk to anyone about receiving it)?

- ○ No (1)
- ○ Yes (2)

Page Break

© 2022 Levy AG et al. *JAMA Network Open*.

MaskWearing How often, if at all, have you worn a protective mask in each of the following places in the past month?

	Never (1)	Some of the time (2)	Most of the time (3)	Every time (4)	I don't go to this place (5)
Outdoors in crowded places (1)	○	○	○	○	○
Outdoors with friends and family (2)	○	○	○	○	○
At work (3)	○	○	○	○	○
In a retail or grocery store (4)	○	○	○	○	○
On public transportation (5)	○	○	○	○	○

SocialNorm Would you say that you are generally taking COVID-19 prevention measures (e.g., wearing protective masks, social distancing) more or less than the people you interact with?

○ Much less (1)

○ Somewhat less (2)

○ About the same (3)

○ Somewhat more (4)

○ Much more (5)

© 2022 Levy AG et al. *JAMA Network Open*.

MaskMandateSchools Are the public schools in your community requiring (mandating) protective masks?

- No (1)
- Yes (2)
- Unsure (3)

Page Break

© 2022 Levy AG et al. *JAMA Network Open*.

VaxMandateWork Has your employer/work required (mandated) that you be vaccinated for COVID-19?

- ○ I am not currently employed (1)
- ○ No (2)
- ○ Yes: My employer requires that everyone be vaccinated (3)
- ○ Yes: My employer requires that everyone be vaccinated OR get regular COVID-19 testing (4)

VaxRequireOther Besides work, is there any place that you have wanted to go that requires people to be vaccinated for COVID-19 (e.g., place of worship, college, theater, sports stadium, restaurant, cruise)?

- ○ No (1)
- ○ Yes (2)

Page Break

© 2022 Levy AG et al. *JAMA Network Open*.

MedExempt Do you have a medical reason for not getting a COVID-19 vaccine?

- No (1)
- Yes (2)

ReligExempt Do you have a religious reason for not getting a COVID-19 vaccine?

- No (1)
- Yes (2)

Display This Question:

If MedExempt = 2

MedExemptOpen Please describe your medical reason for not getting a COVID-19 vaccine.

____

____

____

____

____

Display This Question:

If ReligExempt = 2

ReligExemptOpen Please describe your religious reason for not getting a COVID-19 vaccine.

____

____

____

____

© 2022 Levy AG et al. *JAMA Network Open*.

Page Break

© 2022 Levy AG et al. *JAMA Network Open*.

Vax_FamFrnd About how many of your friends and family do you think have been vaccinated?

- ○ None of them (1)
- ○ A few of them (2)
- ○ Some of them (3)
- ○ Most of them (4)
- ○ All of them (5)

Page Break

© 2022 Levy AG et al. *JAMA Network Open*.

AnyThoughts Use the space below to describe your thoughts about the questions you have answered so far, or if you want to explain any of your answers.

____

____

____

____

____

End of Block: Vax and Masking

Start of Block: Demographics

Gender What is your gender?

- Female (1)
- Male (2)
- Transgender woman /Transwoman (3)
- Transgender man /Transman (4)
- Non-binary/third gender (5)
- Prefer to self-describe (6) ____
- Prefer to not say (7)

Age What is your age?

____

© 2022 Levy AG et al. *JAMA Network Open*.

Page Break

© 2022 Levy AG et al. *JAMA Network Open*.

Race What is your race? Mark all that apply

- ☐ American Indian or Alaskan Native (1)
- ☐ Asian or Asian American (2)
- ☐ Black or African American (3)
- ☐ Native Hawaiian or other Pacific Islander (4)
- ☐ White or European American (5)
- ☐ Other (please specify): (6) ____

Hispanic Are you Hispanic or Latino/a or Latinx?

- ○ No (1)
- ○ Yes (2)

Page Break

© 2022 Levy AG et al. *JAMA Network Open*.

Education What is the highest level of schooling you have completed?

- None (1)
- Elementary school (2)
- Some high school but no diploma (3)
- High school (Diploma or GED) (4)
- Some college, but no degree (5)
- Trade school (6)
- Bachelor's degree (BS, BA, etc.) (7)
- Master's degree (MA, MPH, etc.) (8)
- Doctoral/Professional degree (PhD, MD, etc.) (9)

UrbanRural How would you best describe the place where you live?

- Rural (1)
- Small city (e.g., less than 100,000 people) (2)
- Suburban, near a large city (3)
- Mid-sized city, 100,000 to 1 million people (4)
- Large city, more than 1 million (5)
- Other, please specify: (6) ____

© 2022 Levy AG et al. *JAMA Network Open*.

State What state do you live in?

▼ Alabama (1) ... Wyoming (52)

Page Break

© 2022 Levy AG et al. *JAMA Network Open*.

Political Which political party are you affiliated with?

- Democrat (1)
- Republican (2)
- Independent (3)
- Liberal third party (4)
- Conservative third party (5)
- No political party affiliation (6)

PoliticalSocial How would you describe your political outlook with regard to *social* issues?

- Very liberal (1)
- Liberal (2)
- Slightly liberal (3)
- Moderate (4)
- Slightly conservative (5)
- Conservative (6)
- Very conservative (7)

© 2022 Levy AG et al. *JAMA Network Open*.

Religiosity How religious are you?

- ○ Not at all religious 1 (1)
- ○ 2 (2)
- ○ 3 (3)
- ○ 4 (4)
- ○ 5 (5)
- ○ 6 (6)
- ○ Very religious 7 (7)

ReligiousViews How would you describe your religious views?

- ○ Very traditional 1 (1)
- ○ 2 (2)
- ○ 3 (3)
- ○ 4 (4)
- ○ 5 (5)
- ○ 6 (6)
- ○ Very progressive 7 (7)
- ○ I'm not religious at all 8 (8)

End of Block: Demographics

Start of Block: Individual differences

© 2022 Levy AG et al. *JAMA Network Open*.

VaxGeneral How do you feel about vaccines, in general?

- Very negative (1) (1)
- (2) (2)
- (3) (3)
- (4) (4)
- (5) (5)
- (6) (6)
- Very positive (7) (7)

© 2022 Levy AG et al. *JAMA Network Open*.

EVCI1 Please rate your level of trust in each of the following items:

	No trust (1)	Little trust (2)	Moderate trust (3)	Complete trust (4)	Don't know (5)
Scientists involved in developing and testing new vaccines (1)	○	○	○	○	○
Federal government agencies responsible for monitoring the safety of recommended vaccines (2)	○	○	○	○	○
Centers for Disease Control and Prevention (CDC), the federal government agency that makes recommendations about who should get licensed vaccines (3)	○	○	○	○	○
Food & Drug Administration (FDA), the federal government agency that licenses vaccines (4)	○	○	○	○	○

Page Break

© 2022 Levy AG et al. *JAMA Network Open*.

EVCI2 Please indicate your level of confidence in each item below.

	Not confident at all (1)	Somewhat confident (2)	Confident (3)	Mostly confident (4)	Very confident (5)	I don't know (6)
Vaccines recommended for children are safe. (EVCI2_1)	○	○	○	○	○	○
Vaccines recommended for adults are safe. (EVCI2_2)	○	○	○	○	○	○
My doctor/nurse is a reliable source of trustworthy vaccine information. (EVCI2_3)	○	○	○	○	○	○
My doctor/nurse has my best health interests in mind when making vaccine recommendations. (EVCI2_4)	○	○	○	○	○	○

© 2022 Levy AG et al. *JAMA Network Open*.

EVCI3 Indicate your agreement or disagreement with the following statements:

	Strongly disagree (1)	Disagree (2)	Neutral (3)	Agree (4)	Strongly agree (5)
It is important for everyone to get the recommended vaccines for them (EVCI3_1)	○	○	○	○	○
It is important for everyone to get the recommended vaccines for their child(ren) (EVCI3_2)	○	○	○	○	○

Page Break

© 2022 Levy AG et al. *JAMA Network Open*.

© 2022 Levy AG et al. *JAMA Network Open*.

beliefinscience Please respond to the following statements. Indicate how much you agree or disagree with each statement. There are no right or wrong answers. Please answer in a way that reflects your own personal beliefs.

	Strongly disagree (1)	Disagree (2)	Somewhat disagree (3)	Neither agree nor disagree (4)	Somewhat agree (5)	Agree (6)	Strongly agree (7)
People trust scientists a lot more than they should (beliefinscience_1)	○	○	○	○	○	○	○
People don't realize just how flawed a lot of scientific research really is (beliefinscience_2)	○	○	○	○	○	○	○
A lot of scientific theories are dead wrong (beliefinscience_3)	○	○	○	○	○	○	○
Sometimes I think we put too much faith in science (beliefinscience_4)	○	○	○	○	○	○	○
Our society places too much emphasis on science (beliefinscience_5)	○	○	○	○	○	○	○
I am concerned by the amount of influence that scientists have in society (beliefinscience_6)	○	○	○	○	○	○	○

© 2022 Levy AG et al. *JAMA Network Open*.

Page Break

© 2022 Levy AG et al. *JAMA Network Open*.

conspiracy Below are things that some people might believe. Please indicate whether you personally think each statement is true or false.

© 2022 Levy AG et al. *JAMA Network Open*.

	Definitely false (1)	Probably false (2)	Unsure (3)	Probably true (4)	Definitely true (5)
The virus causing COVID-19 was purposefully released by a government or person. (conspiracy_1)	○	○	○	○	○
COVID-19 is actually a biological weapon being tested. (conspiracy_2)	○	○	○	○	○
The current COVID-19 outbreak is actually a form of population control to reduce the number of people in the infected countries. (conspiracy_3)	○	○	○	○	○
The COVID-19 vaccine has a microchip so the government can track you. (conspiracy_4)	○	○	○	○	○
COVID-19 is not real (conspiracy_5)	○	○	○	○	○

© 2022 Levy AG et al. *JAMA Network Open*.

The COVID-19 vaccine causes infertility (difficulty having children) (conspiracy_6)	○	○	○	○	○

Page Break

© 2022 Levy AG et al. *JAMA Network Open*.

InfoSources From where do you get guidance regarding COVID-19?

	Yes (2)	No (1)
My doctor (InfoSources_1)	○	○
Friends (InfoSources_2)	○	○
Family (InfoSources_3)	○	○
Local Department of Health (InfoSources_4)	○	○
Centers for Disease Control and Prevention (CDC) (InfoSources_5)	○	○
Google (InfoSources_6)	○	○
A certain politician (If yes, please provide the person's name:) (InfoSources_7)	○	○
A certain celebrity (If yes, please provide the person's name:) (InfoSources_8)	○	○
A certain media personality (If yes, please provide the person's name:) (InfoSources_11)	○	○

End of Block: Individual differences

Start of Block: Debrief

Debrief Thank you for your participation! You are now finished with this survey.

In this study, we are interested in understanding the physical, medical, and social impacts that the COVID-19 pandemic has had on members of the public. We greatly appreciate all of your responses! We are learning a lot about how people are responding to and coping with the

© 2022 Levy AG et al. *JAMA Network Open*.

coronavirus pandemic, and we hope to share our findings broadly soon.

If you would like to obtain accurate, up-to-date information about COVID-19, visit the CDC website: https://www.cdc.gov/coronavirus/2019-ncov/index.html

Thank you for participating in this survey.

End of Block: Debrief

© 2022 Levy AG et al. *JAMA Network Open*.
